# Orchestration of kaempferol nuclear translocation by phase ii metabolic enzymes and efflux transporters suppresses colorectal cancer

**DOI:** 10.1371/journal.pone.0355962

**Published:** 2026-09-15

**Authors:** Chishun Zhou, Qing Ning, Xin Jin, Yu Li, Yuefang Lin, Jiarun Lin, Jing Zheng, Yukai Huang, Wei Wang, Zhongqiu Liu, Linlin Lu

**Affiliations:** 1 Chinese Medicine Guangdong Laboratory (Hengqin Laboratory), Guangdong Hengqin, China; and Guangdong Provincial Key Laboratory of Translational Chinese Medicine, Joint International Research Laboratory of Translational Cancer Research of Chinese Medicines, State Key Laboratory of Traditional Chinese Medicine Syndrome, State Key Laboratory of Dampness Syndrome of Chinese Medicine, International Institute for Translational Chinese Medicine, School of Pharmaceutical Sciences, Guangzhou University of Chinese Medicine, Guangzhou, China; 2 Affiliated Hospital of Integrated Traditional Chinese and Western Medicine, Nanjing University of Chinese Medicine, Nanjing, China; 3 Department of Colorectal Surgery, The First Affiliated Hospital of Guangzhou University of Chinese Medicine, Guangdong Clinical Research Academy of Chinese Medicine, Guangzhou, China; 4 Department of Gastrointestinal Surgery, The First Affiliated Hospital of Guangzhou University of Chinese Medicine, Guangdong Clinical Research Academy of Chinese Medicine, Guangzhou, China; Bai Jerbai Wadia Hospital for Children, INDIA

## Abstract

Kaempferol exhibits promising anti-colorectal cancer (CRC) activity, but its intracellular exposure and efficacy are strongly influenced by phase II metabolism and efflux transport. Here, we show that UGT1A1 and UGT1A9 differentially regulate kaempferol accumulation and anti-CRC activity, and that ABCG2/BCRP and Mdr1/P-gP play distinct roles in its intracellular retention and pharmacological effects. Anti-CRC activity was evaluated using sulforhodamine B, colony formation, wound-healing, Transwell, apoptosis, and xenograft assays. Knockdown models of UGT1A1/UGT1A9 and BCRP/P-gP were combined with laser scanning confocal microscopy and LC/MS to characterize intracellular exposure, metabolite disposition, and functional consequences. SIP-based proteomics, bioinformatic analyses, molecular docking, and orthogonal validation assays were used to prioritize and validate a nuclear target of kaempferol. Kaempferol significantly suppressed tumour growth and inhibited CRC cell proliferation and migration. UGT1A1 knockdown increased intracellular accumulation of kaempferol, including nuclear exposure, but reduced cytotoxicity, whereas UGT1A9 knockdown enhanced cellular sensitivity. Similarly, BCRP knockdown increased intracellular accumulation and anti-CRC efficacy, whereas Mdr1 knockdown attenuated the antitumour effect despite increasing total intracellular accumulation. Mechanistically, these differential effects were associated with altered metabolite composition, particularly the balance between the active metabolite K-7-G and the weakly active metabolite K-3-G. BRG1 was prioritized as a functionally relevant nuclear target of kaempferol, and kaempferol promoted its ubiquitination-dependent proteasomal degradation. Together, these findings show that kaempferol efficacy is determined not simply by intracellular accumulation, but by the coordinated interplay among phase II metabolism, efflux transport, metabolite disposition, and nuclear target engagement. This study provides a mechanistic basis for improving kaempferol-based CRC therapy.

## Introduction

The absorption, metabolism, and intracellular disposition of small molecules are major determinants of therapeutic efficacy and safety. Flavonoids are a large class of dietary polyphenols with diverse pharmacological activities, including anticancer effects [1]. However, their clinical translation is often limited by poor bioavailability. After intestinal absorption, flavonoids undergo extensive phase II metabolism, mainly mediated by UDP-glucuronosyltransferases (UGTs), sulfotransferases (SULTs), and glutathione S-transferases (GSTs), which convert them into more hydrophilic conjugates and thereby alter membrane permeability, tissue distribution, and pharmacological activity [2,3]. In parallel, efflux transporters such as P-glycoprotein (P-gP), breast cancer resistance protein (BCRP), and multidrug resistance-associated proteins (MRPs) regulate the export of parent compounds and their conjugates, further shaping intracellular exposure [4,5]. Thus, the coordinated actions of phase II metabolic enzymes and efflux transporters are central determinants of flavonoid disposition.

Colorectal cancer (CRC) remains one of the most common gastrointestinal malignancies worldwide. Current treatment strategies, including surgery, chemotherapy, radiotherapy, and targeted therapy, are limited by drug resistance, adverse effects, and marked interpatient variability [6]. Flavonoids have shown considerable promise in CRC prevention and treatment [7,8], yet their low bioavailability remains a major obstacle to clinical application. Previous studies have shown that transporter deficiency can markedly alter flavonoid exposure; for example, the oral bioavailability of daidzein and its conjugates is substantially increased in Bcrp1-deficient mice [9]. These observations highlight the importance of the interplay between metabolism and efflux transport in determining flavonoid efficacy.

Kaempferol is a naturally occurring flavonol and one of the major active constituents of *Kaempferia galanga Linn*. Increasing evidence indicates that kaempferol exerts inhibitory effects against CRC [10,11]. However, its systemic bioavailability is low [12,13], whereas its antitumour activity remains evident, suggesting that tissue-level and subcellular exposure may be more relevant than plasma concentration alone. Kaempferol is predominantly metabolized by UGT1A1 and UGT1A9 to form kaempferol-7-glucuronide and kaempferol-3-glucuronide, respectively [12,14]. Its cellular efflux is mainly regulated by BCRP and P-gP. Despite these advances, it remains unclear how specific phase II metabolic enzymes and efflux transporters jointly influence kaempferol accumulation, nuclear target engagement, and anti-CRC efficacy.

In the present study, we investigated the anti-CRC activity of kaempferol and the mechanisms governing its intracellular disposition using integrated in vivo and in vitro approaches. We evaluated the respective roles of UGT1A1 versus UGT1A9 and BCRP versus P-gP in regulating kaempferol accumulation and activity, and further identified BRG1 as a functionally relevant nuclear target degraded by kaempferol. These findings clarify how phase II metabolism and efflux transport jointly determine the pharmacological effects of kaempferol and provide a mechanistic basis for improving kaempferol-based strategies for CRC treatment.

## Materials and methods

### Cells and Animals

Cells: Human colorectal cancer (CRC) cell lines HCT116 (C1125), Caco-2 (C1115), HCT15 (C1096), RKO (C1226), DLD-1 (C1073), SW480 (C1128), LoVo (C1019), SW620 (C1160), and HT-29 (C1007) were obtained from Shanghai Whelab Bioscience Limited and authenticated by short tandem repeat (STR) profiling. Human normal colon epithelial cells NCM460 (C1227) were obtained from the same vendor and similarly authenticated. Cells were cultured in DMEM (cat. no. 11965092) or RPMI-1640 medium (cat. no. 11875093) supplemented with 10% fetal bovine serum (FBS; cat. no. 10099141C) and 1% penicillin-streptomycin (cat. no. 15140122) (all from Gibco; Thermo Fisher Scientific, Inc.) at 37°C in a humidified incubator containing 5% CO_2_. For experimental design, different CRC cell lines were used according to their biological characteristics.

Animals and ethics statement: Male BALB/c nude mice (3−4 weeks old, 16−18 g) were obtained from the Guangdong Provincial Medical Laboratory Animal Center. All animal experiments were conducted in accordance with the Guide for the Care and Use of Laboratory Animals (National Institutes of Health, USA) and were approved by the Laboratory Animal Ethics Committee of Guangzhou University of Chinese Medicine (permit no. SYXK(Yue) 2024−0144). Mice were housed in a specific pathogen-free (SPF) facility under controlled environmental conditions (22 ± 1°C, 55 ± 5% humidity, 12 h light/dark cycle) with free access to sterilized food and water. All personnel involved in the animal experiments received appropriate training in animal care and handling in accordance with institutional guidelines, and all efforts were made to minimize animal suffering throughout the study.

### In vivo xenograft model

A subcutaneous xenograft model was established by injecting 200 μL phosphate-buffered saline (PBS) containing 5 × 10^5^ HCT116 human colorectal cancer cells into the right flank of 32 four-week-old male BALB/c nude mice. When the tumor volume reached approximately 100 mm^3^, calculated as V = longest diameter × shortest diameter^2^ × 0.52, the mice were randomly assigned to four groups (n = 8 per group) using a random number table. The control group received an equivalent volume of vehicle by daily oral gavage, whereas the low-, medium-, and high-dose groups received kaempferol (MeilunBio, MB2171; purity > 98%) at 50, 100, and 150 mg/kg, respectively, by daily oral gavage.

Humane endpoints were applied throughout the experiment to minimize animal suffering. Mice were monitored daily for general health status, behavior, tumor growth, and body weight. Tumor size was measured using vernier calipers. Predefined euthanasia criteria included: (1) tumor volume exceeding 1000 mm^3^; (2) tumor ulceration or necrosis; (3) body weight loss greater than 20% of baseline body weight; or (4) signs of severe distress, including hunched posture, lethargy, reduced mobility, ruffled fur, or inability to access food or water. Mice meeting any of these criteria were euthanized immediately under isoflurane anesthesia followed by cervical dislocation.

The experiment was terminated on day 19, when the average tumor volume in the control group exceeded 1000 mm^3^ and thus reached the predefined ethical endpoint. After euthanasia, blood samples were collected by cardiac puncture, and serum was separated by centrifugation. Tumors and major organs, including the heart, liver, spleen, lungs, and kidneys, were harvested. Portions of tumor tissues were fixed in 4% paraformaldehyde for histological and immunohistochemical analyses, whereas the remaining tissues were snap-frozen in liquid nitrogen and stored at −80°C for Western blot and quantitative PCR (QPCR) analyses. No animals died before reaching the predefined humane endpoints during the study period.

### Immunohistochemical detection of Ki67/PCNA expression levels in subcutaneously transplanted tumor tissues

Tumor tissues were fixed in 4% paraformaldehyde (Beyotime, P0099-100 mL), dehydrated through a graded ethanol series, cleared in xylene, embedded in paraffin, and sectioned at a thickness of 7 μm. For immunohistochemical staining, tissue sections were deparaffinized, rehydrated, and subjected to microwave-based antigen retrieval. After blocking, the sections were incubated overnight at 4°C with primary antibodies against Ki67 (Abcam, ab16667; 1:200 dilution) or PCNA (Abcam, ab92552; 1:500 dilution). After washing, the sections were incubated with the secondary antibody (Abcam, ab6721; 1:1,000 dilution) for 1 hour at room temperature. Immunoreactivity was visualized using an SABC reagent kit (Beyotime Biotechnology) with 3,3′-diaminobenzidine (DAB) as the chromogenic substrate, followed by hematoxylin counterstaining (Beyotime, C0107-100 mL). The stained sections were then dehydrated, cleared, mounted, and examined under an upright light microscope (DM500, Leica Microsystems, Germany). Three biological replicates were analyzed for each experimental group.

### SRB assay for detecting cell viability

Cells in the logarithmic growth phase were seeded into 96-well plates at a density of 5 × 10^3^ cells per well and allowed to adhere overnight at 37°C in a humidified incubator containing 5% CO_2_. Cells were then treated with graded concentrations of kaempferol (0–100 μM) for 24, 48, or 72 hours. Unless otherwise indicated, cell viability was assessed using the sulforhodamine B (SRB) assay. At the end of treatment, the culture medium was removed, and the cells were fixed with ice-cold 10% trichloroacetic acid (TCA) for 1 hour at 4°C. The plates were then washed five times with deionized water to remove residual TCA and air-dried at room temperature. Subsequently, cells were stained with 0.4% (w/v) SRB solution in 1% acetic acid (Sigma-Aldrich, cat. no. 341738) for 30 minutes at room temperature. Unbound dye was removed by washing the plates four times with 1% acetic acid, followed by air-drying. Protein-bound SRB was dissolved in 10 mM Tris-base buffer (pH 10.5) with orbital shaking at 200 rpm for 15 minutes. Absorbance was measured at 540 nm with background subtraction at 690 nm using a multimode microplate reader (Synergy H1, BioTek Instruments). Cell viability was expressed as a percentage relative to the untreated control group. IC50 values were calculated from SRB dose-response curves by nonlinear regression analysis using GraphPad Prism software. Six technical replicates were included for each group, and each experiment was performed in three independent biological replicates.

### Transwell assay to evaluate cell migration ability

Cell migration was assessed using Transwell chambers. HCT116 cells were seeded into the upper chambers and allowed to attach overnight. The culture medium was then replaced with fresh medium containing different concentrations of kaempferol, and the cells were further incubated for 48 hours. At the end of the incubation period, cells remaining on the upper surface of the membrane were gently removed. Cells that had migrated to the lower surface were fixed with 4% paraformaldehyde, washed with PBS, and stained with crystal violet. After excess dye was removed, migrated cells were observed and photographed under an upright light microscope (DM500, Leica Microsystems, Germany). Cell images were analyzed and the number of migrated cells was quantified using ImageJ software. Three independent biological replicates were performed for each group.

### Wound healing assay to evaluate cell migration ability

HCT116 cells were seeded in six-well plates and cultured until they reached 70%−80% confluence. A linear wound was generated in the cell monolayer using a sterile pipette tip. Detached cells were removed by washing twice with PBS, and fresh culture medium containing the indicated concentrations of kaempferol was then added. Wound closure was monitored by capturing images at 0, 12, 24, and 48 hours using an inverted fluorescence microscope (DM IL LED, Leica Microsystems, Germany). Three independent biological replicates were performed for each group.

### Colony formation

HCT116 cells were seeded in six-well plates and cultured for 14 days, with the culture medium replaced every 2 days. At the end of the incubation period, the culture medium was removed, and the cells were washed with PBS, fixed with 4% paraformaldehyde, and air-dried. The colonies were then stained with crystal violet, and excess stain was removed by gentle washing. After drying, the colonies were photographed and counted. Three independent biological replicates were performed for each group.

### Flow cytometry detect apoptosis

HCT116 cells were seeded in six-well plates and allowed to adhere overnight. After treatment with the indicated concentrations of kaempferol for 48 hours, both floating and adherent cells were collected, washed twice with cold PBS, and resuspended in 1 × binding buffer. Apoptosis was assessed using an Annexin V-FITC/PI Apoptosis Detection Kit according to the manufacturer’s instructions. Briefly, cells were incubated with Annexin V-FITC and propidium iodide (PI) in the dark at room temperature for 15 minutes and then analyzed immediately by flow cytometry. Flow cytometric analysis was performed using a BD FACSAria Fusion flow cytometer (BD Biosciences, USA), and the data were analyzed using FlowJo software (version 7.6.1; FlowJo LLC, USA). Early apoptotic cells were defined as Annexin V-positive/PI-negative, late apoptotic cells as Annexin V-positive/PI-positive, and total apoptotic fraction was calculated as the sum of early and late apoptotic cells.

### Q-PCR measure the mRNA expression of the target gene

Total RNA was extracted from colorectal cancer cell lines using TRIzol reagent (Accurate Biology, AG21101) according to the manufacturer’s instructions. Briefly, cells were lysed in TRIzol, followed by chloroform extraction and centrifugation. RNA was then precipitated with isopropanol, washed with 75% ethanol, dissolved in DEPC-treated water, and quantified. First-strand cDNA was synthesized using the Evo M-MLV RT kit (Accurate Biology, AG11601) under the following conditions: 25°C for 10 minutes, 42°C for 15 minutes, 85°C for 5 minutes, and 4°C hold. Quantitative real-time PCR (QPCR) was performed on an ABI QuantStudio 5 system (Thermo Fisher Scientific, USA; software version 1.5.1). The reaction mixtures were prepared according to the manufacturer’s instructions, transferred into 384-well plates, sealed, centrifuged, and amplified under the following cycling conditions: 95°C for 30 seconds, followed by 40 cycles of 95°C for 5 seconds and 60°C for 30 seconds. Melting curve analysis was then performed at 95°C for 15 seconds, 60°C for 1 minute, and 95°C for 15 seconds with continuous signal acquisition. All reactions were performed in technical triplicate, and no-template controls were included to ensure specificity. Amplification efficiency for each primer set was validated using standard curves. Three independent biological replicates were performed for each group. Relative mRNA expression levels were calculated using the 2^(-ΔΔCt)^ method with GAPDH as the endogenous reference gene. In this analysis, ΔCt was defined as the difference between the Ct values of the target gene and GAPDH, and ΔΔCt was defined as the difference between the ΔCt values of the experimental and control groups. Statistical analysis and graphical presentation were performed using GraphPad Prism version 9.0 (GraphPad Software Inc., USA), and group comparisons were conducted using Student’s t-test or one-way ANOVA, as appropriate. (S1 Table).

### Lentiviral transfection to construct knockdown cells

HT-29 and Caco-2 cells were seeded into six-well plates and cultured until they reached approximately 30% confluence. Cells were then transduced with lentiviral vectors carrying shRNAs targeting the genes of interest in fresh culture medium supplemented with Polybrene. After 24 hours, the medium was replaced, and the cells were cultured for an additional 72 hours. Transduction efficiency was preliminarily evaluated by observing GFP fluorescence under a fluorescence microscope. Stable transfected cells were subsequently selected using puromycin. The knockdown efficiency was confirmed by QPCR and Western blot analysis. Monoclonal expansion was then performed to establish stable knockdown cell lines.

### Western Blot experiment to assess the knockdown level of the target protein

Cells were lysed in RIPA buffer (Thermo Scientific™, 89901) supplemented with protease inhibitors. After centrifugation to remove cell debris, the supernatants were collected for protein quantification. Total protein concentrations were determined using a bicinchoninic acid (BCA) protein assay kit (Pierce™, Thermo Fisher Scientific, cat. no. 23225) according to the manufacturer’s instructions, with bovine serum albumin (BSA) as the standard. Absorbance was measured at 562 nm using a multimode microplate reader. Protein samples were adjusted to equal concentrations and mixed with 5 × loading buffer. Equal amounts of total protein (30 μg per lane) were separated on 10% SDS-polyacrylamide gels and then transferred onto PVDF membranes. After blocking with 5% bovine serum albumin (BSA), the membranes were incubated overnight at 4°C with primary antibodies against UGT1A1 (Abcam, ab170858), UGT1A9 (Abcam, ab180707), BCRP (Abcam, ab207732), P-gP (Abcam, ab261736), BRG1, or GAPDH. After washing, the membranes were incubated for 1 hour at room temperature with the appropriate horseradish peroxidase (HRP)-conjugated secondary antibodies diluted in TBST containing 5% skim milk. For rabbit-derived primary antibodies, HRP-conjugated goat anti-rabbit IgG (Invitrogen, cat. no. A27036) was used at a dilution of 1:5,000. For mouse-derived primary antibodies, HRP-conjugated goat anti-mouse IgG (Invitrogen, cat. no. A27012) was used at a dilution of 1:5,000. GAPDH was used as the loading control for normalization. Protein bands were visualized using SuperSignal™ West Pico PLUS Chemiluminescent Substrate (Thermo Fisher Scientific, cat. no. 34580) according to the manufacturer’ s instructions. Three independent biological replicates were performed for each group.

### LC/MS detection of the rate of metabolism of kaempferol in cells [15,16]

Caco-2 and HT-29 cells, including their UGT1A1- and UGT1A9-knockdown variants, were treated with kaempferol under the indicated conditions. After treatment, cells were washed twice with ice-cold PBS and collected for metabolite extraction. Intracellular metabolites were extracted with ice-cold 80% methanol containing the internal standard rutin. The samples were vortexed, sonicated on ice, and centrifuged at 14,000 × g for 15 min at 4°C. The supernatants were collected, dried under a gentle nitrogen stream at 40°C, and reconstituted in 50% acetonitrile containing 0.1% formic acid before LC-MS analysis. For normalization, parallel cell samples were lysed with RIPA buffer, and total protein concentrations were determined using a BCA protein assay kit. For subcellular metabolite analysis, cytoplasmic and nuclear fractions were prepared according to the corresponding fractionation protocol and processed using the same extraction procedure. Metabolite levels were normalized to protein concentration. Kaempferol, kaempferol-7-O-glucuronide (K-7-G), and kaempferol-3-O-glucuronide (K-3-G) were analyzed using an ultra-high-performance liquid chromatography system coupled to a Xevo G2-XS Q-TOF mass spectrometer. Chromatographic separation was performed on an ACQUITY UPLC BEH C18 column using 0.1% formic acid in water as mobile phase A and 0.1% formic acid in acetonitrile as mobile phase B. The flow rate was 0.3 mL/min. Mass spectrometric detection was conducted in negative electrospray ionization mode. Kaempferol was detected as [M-H]- at m/z 285.040, while K-3-G and K-7-G were detected as [M-H]- at m/z 461.073, with a characteristic fragment ion at m/z 285.040. The identification of K-3-G and K-7-G was based on accurate mass, MS/MS fragmentation, retention time, and comparison with authentic standards.

### SIP (Solvent-Induced Protein Precipitation) and CETSA (Cellular Thermal Shift Assay) assay for screening candidate kaempferol-interacting proteins

SIP was used to screen candidate proteins potentially directly interacting with kaempferol. The principle of this assay is that binding of a small-molecule ligand may alter protein conformation and thereby change the susceptibility of the bound protein to precipitation in the presence of an organic solvent mixture. Under the assay conditions used in this study, proteins associated with kaempferol binding were more likely to remain enriched in the supernatant after solvent treatment. Briefly, the protein concentration of the cell lysates was adjusted to 5 mg/mL, and the lysates were divided into two aliquots. One aliquot was incubated with 100 μM kaempferol, whereas the other was incubated with an equal volume of DMSO as the solvent control, for 1 hour at room temperature. A mixed organic solvent system (ethanol:acetone:acetic acid = 50:50:1) was then added to a final concentration of 12%, followed by incubation at room temperature for 30 minutes (for CETSA, heat at a gradient temperature from °C to 65°C for 5 minutes). Samples were centrifuged at 4°C for 15 minutes, and the resulting supernatants were collected. The supernatants were mixed with loading buffer, heated at 100°C for 5 minutes, and subjected to polyacrylamide gel electrophoresis. Protein bands were visualized using a rapid silver-staining kit and imaged for analysis. Protein bands enriched in the supernatant of the kaempferol-treated group relative to the DMSO control group were excised and subjected to mass spectrometry identification. In this study, the SIP readout was defined as the relative enrichment of proteins in the supernatant after kaempferol treatment compared with the DMSO control. Accordingly, these proteins were treated as candidate kaempferol-interacting proteins rather than proteins with altered expression levels.

### Bioinformatics analysis

SIP-derived supernatant protein samples were subjected to mass spectrometry-based proteomic analysis by Hangzhou Jingjie Biotechnology Co., Ltd. Proteins enriched in the supernatant of the kaempferol-treated group relative to the DMSO control group were defined as candidate kaempferol-interacting proteins. In total, 62 proteins were identified as enriched candidate proteins, whereas 248 proteins were less enriched in the kaempferol-treated supernatant and were not interpreted as differentially expressed proteins. Subcellular localization analysis of the 62 candidate proteins was performed using the UniProt database (https://www.uniprot.org/). Protein domain annotation was conducted using PfamScan version 1.6 based on the Pfam database (https://pfam.xfam.org/). Gene Ontology (GO) and Kyoto Encyclopedia of Genes and Genomes (KEGG) pathway enrichment analyses were performed for the 62 candidate proteins using the GO database (http://geneontology.org/) and the KEGG database (https://www.genome.jp/kegg/), respectively. Fisher’s exact test was used to evaluate the statistical significance of enrichment results. These enrichment data are shown in Fig 4E and summarized in the corresponding supplementary tables. Protein-protein interaction (PPI) networks were constructed using STRING database version 11.5 (https://string-db.org/) and visualized using Cytoscape version 3.8.2. Based on subcellular localization analysis, 37 of the 62 candidate proteins were identified as nuclear-localized proteins. These 37 nuclear proteins were further subjected to PPI network analysis, from which 15 core-node proteins were identified using a confidence score threshold of ≥ 0.7. For molecular docking analysis, the structure of kaempferol was obtained from the NIH NLM platform and the PubChem database (https://pubchem.ncbi.nlm.nih.gov/), whereas protein structures were obtained from the AlphaFold Protein Structure Database (https://alphafold.ebi.ac.uk/). Structure preprocessing was performed using Julia scripts and BioStructures. Molecular docking was carried out using AutoDock Vina version 1.1.2, LeDock version 1.0, and XDock version 2019. Binding energies were calculated and integrated for target prioritization. The top 10 docking-ranked proteins were then intersected with the 15 PPI core proteins to identify BRG1 (SMARCA4) as the principal candidate target. For clinical relevance analysis, The Cancer Genome Atlas (TCGA) and the Clinical Proteomic Tumor Analysis Consortium (CPTAC) databases were used to analyze BRG1 expression and proteomic validation in colorectal cancer. Cell line authentication information was referenced from the Cellosaurus database (https://www.cellosaurus.org/).

### Cycloheximide (CHX) chase assay

To evaluate BRG1 protein stability, cells were treated with cycloheximide (CHX; 100 μM) in fresh culture medium to block de novo protein synthesis. At the indicated time points (0, 3, 6, 9, 12, 24, 36, and 48 hours), cells were collected and lysed for protein extraction. BRG1 protein levels at each time point were analyzed by Western blot, with GAPDH used as the loading control. Band intensities were quantified, normalized to GAPDH, and fitted using a one-phase exponential decay model to estimate the half-life and degradation rate constant of BRG1.

### Immunofluorescence (IF)

Cells were seeded in confocal dishes (NEST, 801002) and cultured overnight. Following drug treatment, they were washed with PBS, fixed with paraformaldehyde, permeabilized with Triton X-100, and blocked with 2.5% BSA. Subsequently, the cells were incubated with the primary antibody overnight at 4°C, followed by incubation with a fluorescently labeled secondary antibody for 1 hour at room temperature (approximately 25°C) under light-protected conditions. Finally, the nuclei were counterstained with a fluorescence quencher containing DAPI (Beyotime Biotechnology, P0131-25 mL), and the samples were observed under a laser confocal microscope (Laser Scanning Confocal Microscope: Leica TCS SP8 (Leica Microsystems, Germany)).

### Ubiquitination assay

To assess BRG1 ubiquitination, cells were pretreated with MG132 (10 μM) for 4 hours to block proteasomal degradation of ubiquitinated proteins and were then treated with kaempferol (50 μM) for 24 hours. Whole-cell lysates were prepared in non-denaturing lysis buffer supplemented with protease inhibitors. BRG1 was immunoprecipitated using an anti-BRG1 antibody and incubated with Protein A/G agarose beads overnight at 4°C. After extensive washing, the immunoprecipitated complexes were subjected to SDS-PAGE and immunoblotted with an anti-ubiquitin antibody (FK2) to detect ubiquitinated BRG1. Total BRG1 in the immunoprecipitates was detected in parallel and used for normalization.

### Co-immunoprecipitation

To evaluate the association between BRG1 and TRIM21, cells were treated with kaempferol (50 μM) for 24 hours and lysed in non-denaturing lysis buffer. Equal amounts of total protein (500 μg) were incubated overnight at 4°C with anti-BRG1 antibody or anti-TRIM21 antibody in the presence of Protein A/G agarose beads. After washing, the immunoprecipitated complexes were analyzed by Western blot to detect the association between BRG1 and TRIM21. Reciprocal co-immunoprecipitation was performed to further validate the interaction.

### 5-Ethynyl-2’-deoxyuridine (EdU)

Cell proliferation was assessed using a EdU incorporation assay. Cells were seeded into 96-well plates at a density of 5 × 10^3^ cells per well and cultured for 24 hours. EdU was then added to the culture medium at a final concentration of 10 μM, and the cells were incubated for an additional 2 hours. After incubation, the cells were fixed with 4% paraformaldehyde for 15 minutes at room temperature and permeabilized with 0.5% Triton X-100 for 20 minutes at room temperature. The cells were then incubated with the Click-iT® reaction cocktail for 30 minutes in the dark. Nuclei were counterstained with DAPI (1 μg/mL) for 10 minutes. Fluorescence images were acquired, and the percentage of EdU-positive cells was calculated to evaluate cell proliferation.

### Statistical analysis

Statistical analyses were conducted using GraphPad Prism 9.0 for graph production and SPSS 21.0 for data analysis. The data were initially assessed for normal distribution and variance homogeneity. One-way ANOVA was utilized to evaluate intergroup differences in normally distributed data with homogeneous variance. If substantial differences were identified (*P* < 0.05), the Tukey post hoc test was employed for multiple comparisons. For data with non-homogeneous variance, the Welch test was used, followed by the Dunnett T3 test for pairwise comparisons. Non-normally distributed data were analyzed using the Kruskal-Wallis test, and if a significant overall difference was detected (*P* < 0.05), Dunn’s multiple comparison test was applied for pairwise group comparisons. For survival analysis, Kaplan–Meier curves were constructed to visualize overall survival differences, and the Log-rank (Mantel-Cox) test was applied to compare survival between high and low BRG1 expression groups. The hazard ratio (HR) and its 95% confidence interval were further calculated using Cox proportional hazards regression to quantify the association between BRG1 expression and survival outcome. Results are presented as mean ± standard deviation (mean ± SD), with P < 0.05 considered statistically significant. Significance levels in figures are indicated as *P < 0.05, **P < 0.01, and ***P < 0.001.

## Results

### In vivo and in vitro verification of the anti-CRC efficacy of kaempferol

**Fig 1:**
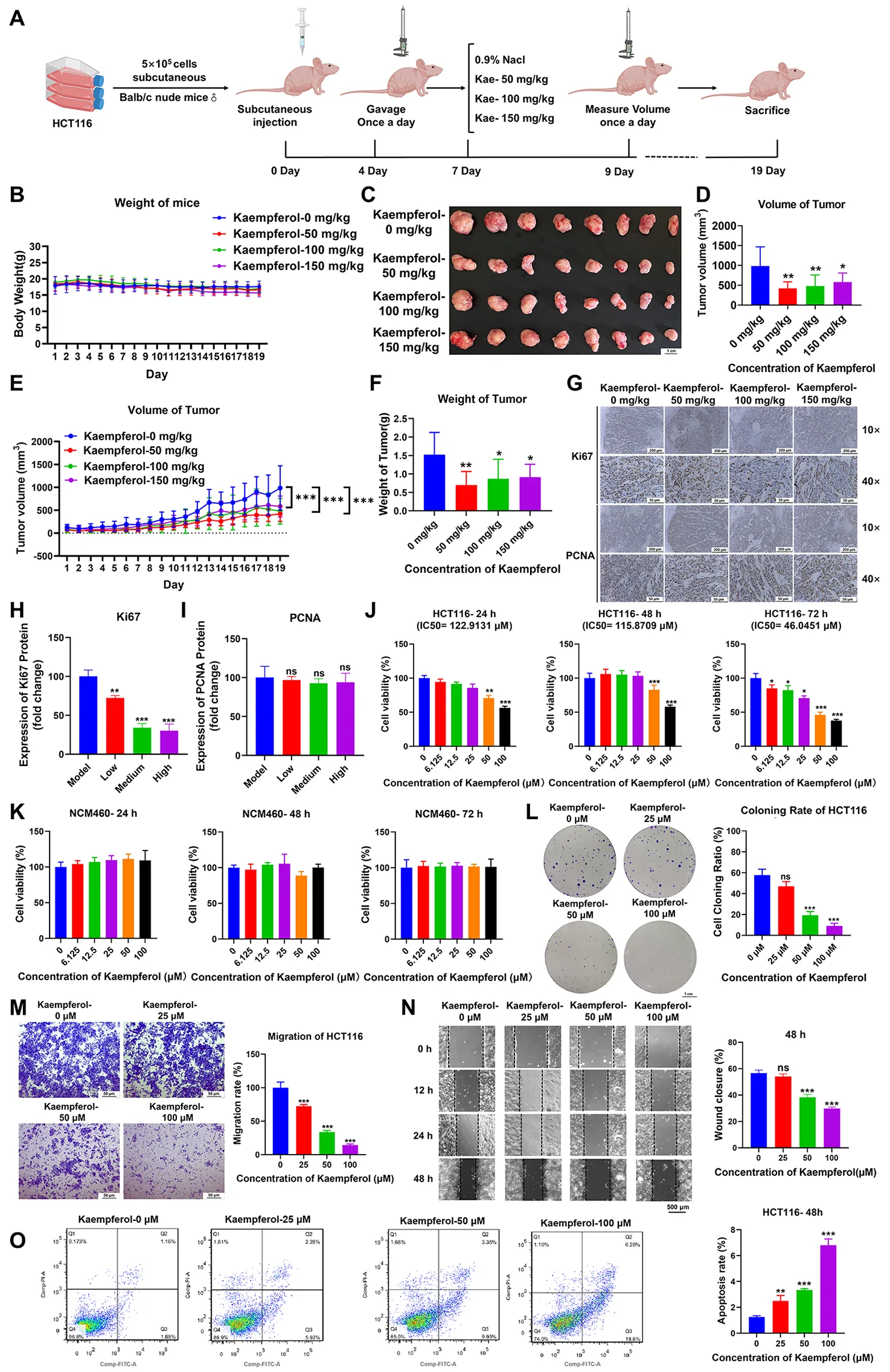
In vivo and in vitro validation of the anti-colorectal cancer activity of kaempferol. (A) Schematic illustration of the HCT116 xenograft model and kaempferol treatment regimen. (B) Changes in body weight of tumor-bearing mice during treatment. (C) Representative images of excised tumors from each group. (D) Final tumor volumes at the endpoint. (E) Tumor growth curves during the treatment period. (F) Final tumor weights at the endpoint. (G) Representative immunohistochemical images of Ki67 and PCNA expression in tumor tissues. (H) Quantification of Ki67 expression. (I) Quantification of PCNA expression. (J) SRB assay showing the effects of kaempferol on the viability of HCT116 cells after 24, 48, and 72 h of treatment. (K) SRB assay showing the effects of kaempferol on the viability of normal colonic epithelial NCM460 cells after 24, 48, and 72 h of treatment. (L) Colony formation assay showing the effect of kaempferol on clonogenic growth of HCT116 cells, with corresponding quantification. (M) Transwell migration assay showing the effect of kaempferol on HCT116 cell migration, with corresponding quantification. (N) Wound-healing assay showing the effect of kaempferol on HCT116 cell migration, with corresponding quantification. (O) Annexin V-FITC/PI flow cytometric analysis of apoptosis in kaempferol-treated HCT116 cells, with corresponding quantification. Data were analyzed by one-way ANOVA. *P < 0.05, **P < 0.01, and ***P < 0.001 versus the corresponding control group.

To evaluate the anti-CRC efficacy of kaempferol, we established a subcutaneous xenograft model using HCT116 cells in mice and administered varying doses of kaempferol (Fig 1A). Kaempferol treatment did not cause significant changes in body weight (Fig 1B), suggesting low systemic toxicity (S1A Fig). Compared with the control group, all kaempferol-treated groups showed significant suppression of tumor growth after 12 days of treatment. At the end of the experiment (day 19), both tumor volume and excised tumor weight were significantly reduced in the kaempferol-treated groups (Fig 1C-F). Immunohistochemical analysis further showed that kaempferol significantly reduced Ki67 expression in tumor tissues (*P* < 0.01 vs. control), whereas no significant difference was observed in PCNA expression (Fig 1G-I).

The sulforhodamine B (SRB) assay showed that kaempferol inhibited the proliferation of HCT116 cells in a concentration- and time-dependent manner (Fig 1J). After 72 h of treatment, 100 μM kaempferol reduced cell viability to 39.34% ± 2.3% (*P* < 0.001). In contrast, no obvious cytotoxicity was observed in normal NCM460 colon epithelial cells at concentrations ≤100 μM (viability > 92%; Fig 1K), indicating a degree of tumor selectivity. Colony formation was also markedly reduced after kaempferol treatment (Fig 1L). Transwell and wound-healing assays consistently showed that kaempferol significantly impaired the migratory capacity of HCT116 cells (Fig 1M-N). In addition, Annexin V-FITC/PI flow cytometric analysis demonstrated that kaempferol induced apoptosis in HCT116 cells in a concentration-dependent manner (*P* < 0.001; Fig 1O), as reflected by increased proportions of early apoptotic, late apoptotic, and total apoptotic cells. Similar antiproliferative and anti-migratory effects were also observed in HT-29 and Caco-2 cells (S1B-D Fig), supporting the cross-cell-line relevance of the anti-CRC activity of kaempferol. Together, these findings demonstrate that kaempferol exerts significant anti-CRC effects both in vivo and in vitro.

### The impact of phase II metabolic enzymes on the anti-CRC efficacy of kaempferol

**Fig 2:**
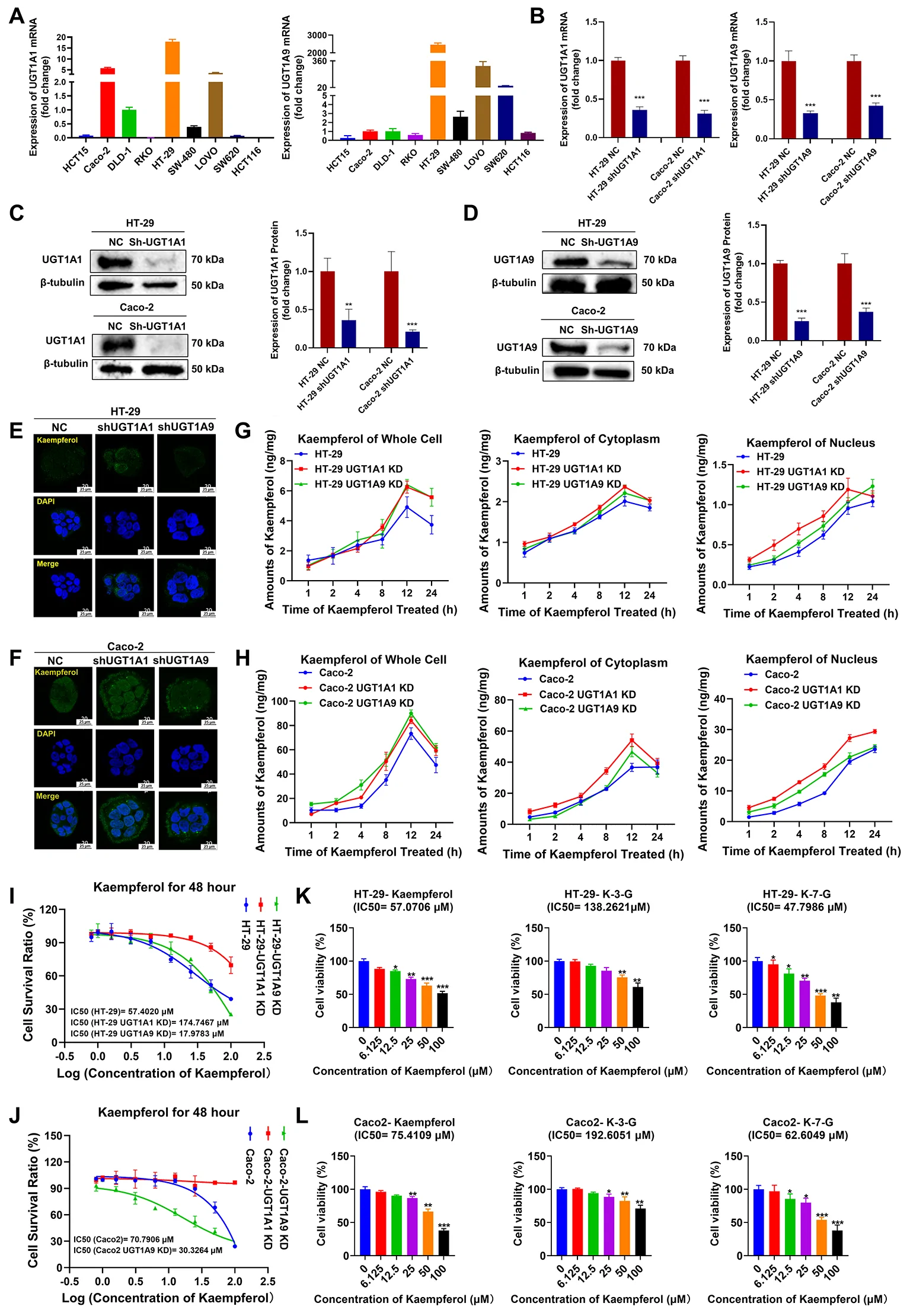
Effects of phase II metabolic enzymes on the anti-colorectal cancer activity of kaempferol. (A) Baseline mRNA expression levels of UGT1A1 and UGT1A9 in different colorectal cancer cell lines. (B) QPCR verification of UGT1A1 and UGT1A9 knockdown in HT-29 and Caco-2 cells. (C) Western blot verification of UGT1A1 knockdown efficiency in HT-29 and Caco-2 cells, with corresponding quantification. (D) Western blot verification of UGT1A9 knockdown efficiency in HT-29 and Caco-2 cells, with corresponding quantification. (E) Laser scanning confocal microscopy (LSCM) analysis of intracellular kaempferol exposure in HT-29 cells. (F) LSCM analysis of intracellular kaempferol exposure in Caco-2 cells. (G) LC-MS/MS quantification of kaempferol in whole-cell lysates, nuclear fractions, and cytoplasmic fractions of HT-29 cells at different time points. (H) LC-MS/MS quantification of kaempferol in whole-cell lysates, nuclear fractions, and cytoplasmic fractions of Caco-2 cells at different time points. (I) SRB assay showing the effects of UGT1A1 or UGT1A9 knockdown on the antiproliferative activity of kaempferol in HT-29 cells. (J) SRB assay showing the effects of UGT1A1 or UGT1A9 knockdown on the antiproliferative activity of kaempferol in Caco-2 cells. (K) Cytotoxic effects of kaempferol and its major metabolites K-7-G and K-3-G in HT-29 cells. (L) Cytotoxic effects of kaempferol and its major metabolites K-7-G and K-3-G in Caco2 cells. Data were analyzed by one-way ANOVA. *P < 0.05, **P < 0.01, and ***P < 0.001 versus the corresponding control group.

Our previous studies identified UGT1A1 and UGT1A9 as the major phase II metabolic enzymes responsible for the conversion of kaempferol to kaempferol-7-glucuronide (K-7-G) and kaempferol-3-glucuronide (K-3-G), respectively [11]. QPCR analysis showed that Caco-2, HT-29, and LoVo cells expressed relatively high levels of UGT1A1 and UGT1A9 (Fig 2A). Knockdown efficiency was confirmed by QPCR and Western blot analyses in both HT-29 and Caco-2 cells (Fig 2B-D). In addition, repeated baseline Western blot analysis performed under matched experimental conditions showed that UGT1A9 protein expression was higher in HT-29 cells than in Caco-2 cells, which was directionally consistent with the QPCR results (S2A Fig). Laser scanning confocal microscopy showed that intracellular kaempferol accumulation increased after UGT1A1 or UGT1A9 knockdown, with the most prominent increase observed in UGT1A1-knockdown cells (Fig 2E-F). LC-MS/MS analysis further showed that intracellular kaempferol exposure in both HT-29 and Caco-2 cells increased over time, reached a maximum at 12 hours, and showed enhanced nuclear accumulation at 24 hours, particularly after UGT1A1 knockdown (Fig 2G-H). Overall intracellular exposure was higher in Caco-2 cells than in HT-29 cells. SRB assays showed that kaempferol exerted time-dependent cytotoxicity in both HT-29 and Caco-2 cells. However, this effect was attenuated after UGT1A1 knockdown, whereas UGT1A9 knockdown increased cellular sensitivity to kaempferol (Fig 2I-J; S2B-E Fig). To further investigate this difference, we compared the activities of the major glucuronidated metabolites. Kaempferol showed significant cytotoxicity at 50.0706 μM, whereas K-7-G exhibited markedly stronger antiproliferative activity, with an effective concentration of 47.7986 μM. In contrast, K-3-G showed little effect on cell viability (Treatment on HT-29 cells for 48 h, Fig 2K) (Results of Caco‑2 are shown in Fig 2L).

To further identify the intracellular active forms with pharmacodynamic relevance, we quantitatively analyzed K‑7‑G and K‑3‑G in the nuclear fraction using LC‑MS/MS. Under identical experimental conditions (treatment with 50 μM kaempferol for 12 h), nuclear lysates were prepared from Caco-2 and HT-29 cells. In Caco-2 cells, the nuclear concentration of K-7-G was 5.8530 ± 0.1738 ng/mg protein, which corresponds to approximately 0.1860‑fold that of the parent kaempferol (38.1767 ± 1.5894 ng/mg protein); the nuclear concentration of K-3-G was 7.6332 ± 0.4467 ng/mg protein, approximately 0.2423‑fold that of the parent compound (S2F Fig). Similar trends were observed in HT-29 cells. Thus, both K-7-G and K-3-G accumulated in the nucleus to markedly lower levels than did the parent kaempferol. Given that K-7-G exhibits superior anti‑CRC activity in vitro but, owing to glucuronidation, has increased polarity and impaired nuclear membrane permeability, these results suggest that the enhanced efficacy of K-7-G may be mediated mainly through extranuclear targets.

### The impact of efflux transporters on the anti-CRC efficacy of kaempferol

**Fig 3:**
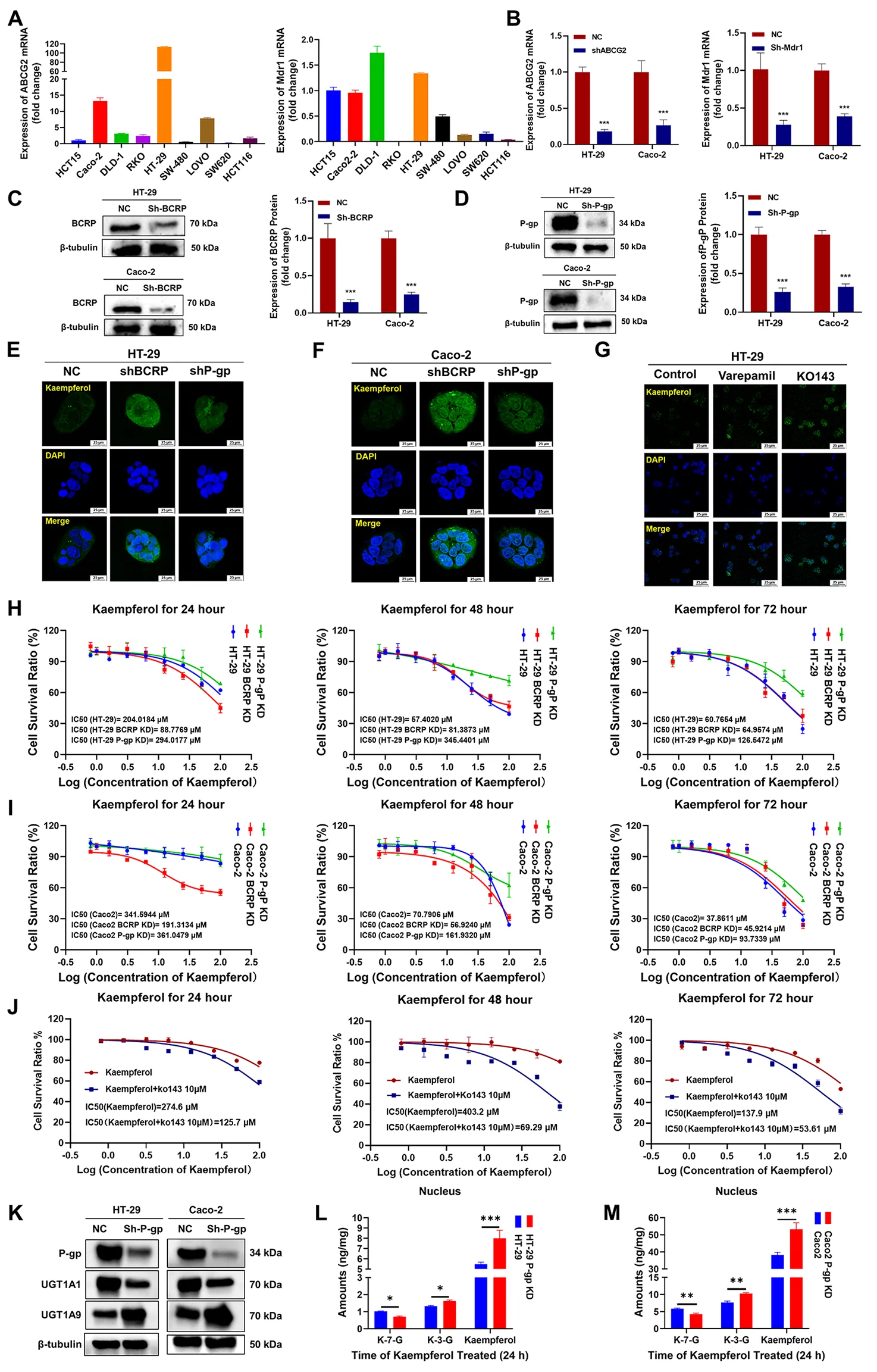
The Influence of Efflux Transporters on the Anti-Colorectal Cancer Efficacy of Kaempferol. (A) Baseline mRNA expression levels of ABCG2 and Mdr1 in different colorectal cancer cell lines. (B) QPCR verification of ABCG2 and Mdr1 knockdown efficiency in transfected cells. (C) Western blot verification of BCRP knockdown efficiency, with corresponding quantification. (D) Western blot verification of P-gP knockdown efficiency, with corresponding quantification. (E) Laser scanning confocal microscopy (LSCM) analysis of intracellular kaempferol exposure in HT-29 cells after BCRP or P-gP knockdown. (F) LSCM analysis of intracellular kaempferol exposure in Caco-2 cells after BCRP or P-gP knockdown. (G) LSCM analysis of the effects of efflux transporter inhibitors on intracellular kaempferol accumulation. (H) SRB assay showing the effects of BCRP or P-gP knockdown on the antiproliferative activity of kaempferol in HT-29 cells. (I) SRB assay showing the effects of BCRP or P-gP knockdown on the antiproliferative activity of kaempferol in Caco-2 cells. (J) SRB assay showing the effect of the BCRP inhibitor KO143 on the antiproliferative activity of kaempferol. (K) P‑gp knockdown upregulates UGT1A9 and downregulates UGT1A1 protein expression in Caco‑2 and HT‑29 cells. (L) Effect of P-gP knockdown on nuclear K-7-G and K-3-G levels in HT-29 cells. (M) Effect of P-gP knockdown on nuclear K-7-G and K-3-G levels in Caco-2 cells. Data were analyzed by one-way ANOVA. *P < 0.05, **P < 0.01, and ***P < 0.001 versus the corresponding control group.

To investigate the contribution of efflux transporters to kaempferol activity, we first examined the baseline expression of ABCG2 (encoding breast cancer resistance protein, BCRP) and Mdr1 (encoding P-glycoprotein, P-gP) in nine colorectal cancer cell lines. Caco-2 and HT-29 cells were selected for further study because both cell lines showed relatively high expression of these transporters (Fig 3A). Stable BCRP- and P-gP-knockdown cell models were then generated by shRNA transfection. Knockdown efficiency was confirmed by QPCR and Western blot analyses, which showed marked reductions in the mRNA and protein levels of BCRP and P-gP in both cell lines (Fig 3B-D). Laser scanning confocal microscopy showed that knockdown of either BCRP or P-gP increased intracellular kaempferol exposure in both HT-29 and Caco-2 cells, as indicated by enhanced fluorescence intensity. This effect was more pronounced after BCRP knockdown (Fig 3E-F). Similarly, pharmacological inhibition of efflux transporters further increased intracellular retention of kaempferol, with the strongest effect observed after treatment with the BCRP inhibitor KO143 (Fig 3G). SRB assays showed that kaempferol exerted comparable cytotoxic effects in HT-29 and Caco-2 cells and that the inhibitory effect increased with both concentration and treatment time. After 72 hours of exposure to 100 μM kaempferol, both cell lines showed approximately 70% growth inhibition. Notably, BCRP knockdown enhanced the antiproliferative effect of kaempferol, whereas P-gP knockdown unexpectedly attenuated it (Fig 3H-I). Consistently, co-treatment with the BCRP inhibitor KO143 further strengthened the inhibitory effect of kaempferol on CRC cell proliferation (Fig 3J). To explain the unexpected reduction in efficacy after P-gP knockdown, we further examined whether P-gP silencing affected the expression of kaempferol-metabolizing enzymes. Western blot analysis showed that P-gP knockdown markedly increased UGT1A9 protein expression by approximately 1.5- to 2.0-fold, while modestly decreasing UGT1A1 expression by approximately 25%−40% in both Caco-2 and HT-29 cells (Fig 3K). Given that UGT1A1 generates the highly active metabolite K-7-G, whereas UGT1A9 mainly produces the weakly active metabolite K-3-G, these results suggest a shift in metabolic flux away from the active form. Consistently, LC-MS/MS analysis showed that although total intracellular kaempferol-related compounds increased after P-gP knockdown, the absolute concentration of K-7-G decreased from 1.0155 ± 0.0251 to 0.7036 ± 0.0342 ng/mg, whereas K-3-G increased from 1.3236 ± 0.0376 to 1.6180 ± 0.0659 ng/mg (Fig 3L; The same trend was observed in Caco‑2 cells, Fig 3M). Taken together, these findings demonstrate that BCRP and P-gP exert divergent effects on kaempferol pharmacology. Whereas BCRP knockdown enhances kaempferol efficacy mainly by increasing intracellular retention of active species, P-gP knockdown reduces efficacy despite increasing total intracellular accumulation, because it redirects metabolic flux toward the less active metabolite.

### Identification of candidate kaempferol-binding proteins and prioritization of BRG1

**Fig 4:**
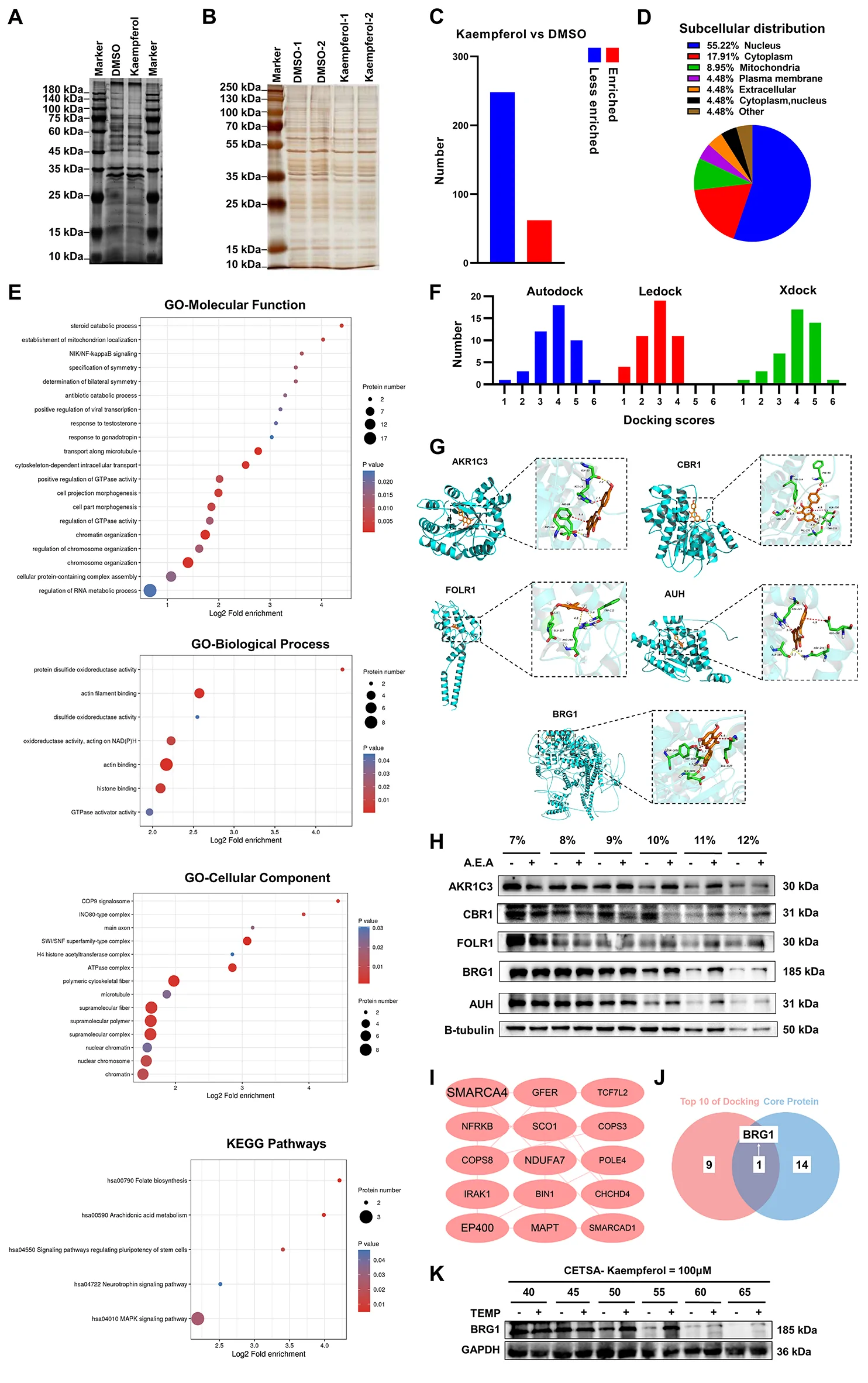
Identification of Kaempferol’s Anti-Colorectal Cancer Targets. (A) Silver-staining results of SIP samples. (B) Quality control analysis of SIP samples. (C) Quantification of proteins differentially enriched in the SIP supernatant between the kaempferol-treated group and the DMSO control group. (D) Subcellular localization of the 62 candidate proteins enriched in the kaempferol-treated supernatant. (E) GO enrichment and KEGG pathway analyses of the 62 candidate kaempferol-interacting proteins. (F) Integrated ranking of molecular docking scores obtained from three docking programs. (G) Representative molecular docking models of five candidate target proteins. (H) SIP-based validation of candidate proteins enriched in the kaempferol-treated supernatant. (I) Protein-protein interaction (PPI) network of nuclear-localized candidate proteins. (J) Intersection analysis between the top 10 docking-ranked proteins and the 15 PPI core proteins. (K) CETSA confirms the direct interaction between kaempferol and BRG1.

Silver staining showed multiple differential protein bands in the supernatant of the kaempferol-treated group compared with the DMSO control group (Fig 4A-B). Quantitative proteomic analysis identified 62 proteins enriched in the kaempferol-treated supernatant, which were therefore defined as candidate kaempferol-interacting proteins, whereas 248 proteins were less enriched in the kaempferol-treated supernatant (Fig 4C; S2 Table). Subcellular localization analysis of the 62 candidate proteins showed that 37 proteins (55.22%) were localized in the nucleus, 12 (17.91%) in the cytoplasm, 6 (8.95%) in the mitochondria, and the remainder in other compartments, including the cell membrane and endoplasmic reticulum (Fig 4D). GO and KEGG enrichment analyses indicated that these candidate proteins were associated with multiple pathways related to cell growth and proliferation, including folate biosynthesis, arachidonic acid metabolism, signaling pathways regulating stem cell pluripotency, and the MAPK signaling pathway (Fig 4E). To further prioritize candidate targets, molecular docking was performed using AutoDock, LeDock, and XDock, and the docking scores were integrated after linear normalization. This analysis yielded the top 10 docking-ranked proteins, including AKR1C3, CBR1, BRG1, and other candidates with favorable predicted binding energies (Fig 4F; S3-5 Tables). Based on the docking results, five representative candidate proteins were selected for further validation (Fig 4G). SIP validation confirmed that AKR1C3, FOLR1, AUH, BRG1, and CBR1 were consistently enriched in the kaempferol-treated supernatant (Fig 4H). A protein-protein interaction (PPI) network was then constructed for the 37 nuclear-localized candidate proteins, identifying 15 core-node proteins with high connectivity (Fig 4I). Intersection of these 15 PPI core proteins with the top 10 docking-ranked proteins led to the prioritization of BRG1 (SMARCA4) as the principal candidate target (Fig 4J). Finally, the binding between kaempferol and BRG1 was further validated by CETSA (Fig 4K).

### Kaempferol induces BRG1 degradation and suppresses colorectal cancer progression

**Fig 5:**
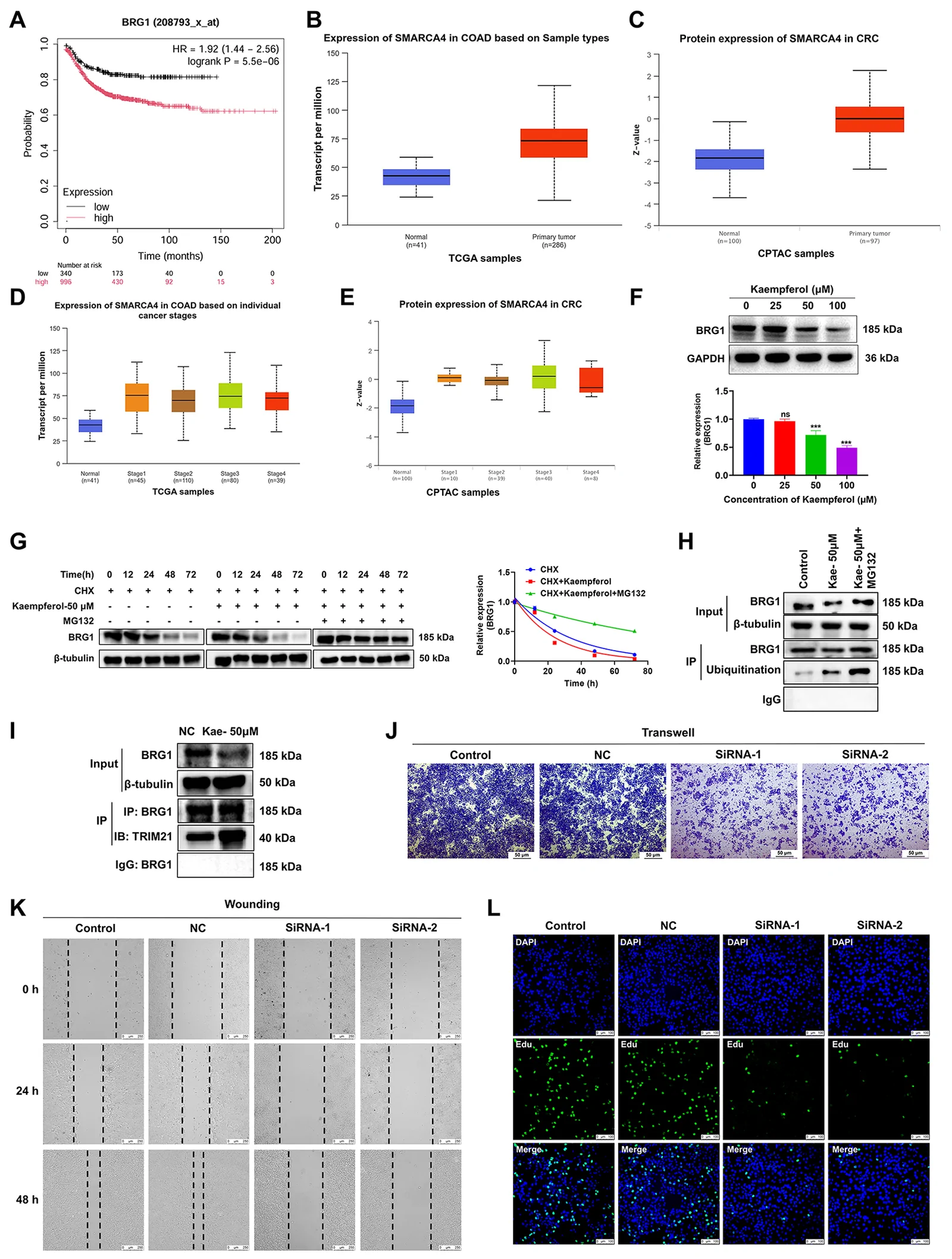
Clinical relevance of BRG1 and kaempferol-induced BRG1 degradation in colorectal cancer cells. (A) Kaplan-Meier survival analysis of CRC patients stratified by BRG1 expression. (B) TCGA analysis of BRG1 mRNA expression in normal colorectal tissues and CRC tissues. (C) CPTAC analysis of BRG1 protein expression in normal colorectal tissues and CRC tissues. (D) TCGA analysis of BRG1 mRNA expression across different CRC stages. (E) CPTAC analysis of BRG1 protein expression across different CRC stages. (F) Western blot analysis of the effect of kaempferol on BRG1 protein abundance, with corresponding quantification. (G) Kaempferol reduces BRG1 protein stability via the proteasome pathway, as shown by CHX chase and MG132 rescue. (H) Ubiquitination assay showing kaempferol-induced ubiquitination of BRG1 following immunoprecipitation. (I) Co-immunoprecipitation assay showing the association between BRG1 and TRIM21 after kaempferol treatment. (J) Transwell migration assay showing the effect of BRG1 knockdown on CRC cell migration. (K) Wound-healing assay showing the effect of BRG1 knockdown on CRC cell migration. (L) EdU incorporation assay showing the effect of BRG1 knockdown on CRC cell proliferation. Data were analyzed by one-way ANOVA. *P < 0.05, **P < 0.01, and ***P < 0.001 versus the corresponding control group.

To explore BRG1’s role in kaempferol-mediated CRC suppression, we analysed clinical Kaplan-Meier survival curves and found that higher BRG1 expression correlates with shorter overall survival in CRC patients (HR = 1.92, *P* < 0.001; Fig 5A). Analyses of TCGA and CPTAC datasets revealed BRG1 is tumour-specifically overexpressed compared to normal tissues (Fig 5B-C). Notably, BRG1 expression increases with tumour progression (Fig 5D-E). Western blot analysis showed that kaempferol reduced BRG1 protein abundance in a dose-dependent manner in colorectal cancer cells. Quantification demonstrated that treatment with 50 μM kaempferol for 24 hours decreased BRG1 protein levels to 52% ± 5% of the control level (*P* < 0.001; Fig 5F). To determine whether kaempferol promotes BRG1 degradation, CHX chase assays were performed and BRG1 decay curves were fitted using a one-phase exponential decay model. In the control group (CHX alone), the half-life of BRG1 was 23.03 hours (95% CI: 18.98–28.25 hours), whereas co-treatment with kaempferol (CHX + kaempferol) shortened the half-life to 17.07 hours (95% CI: 13.61–21.62 hours). To test whether this effect was proteasome-dependent, cells were treated with kaempferol in the presence of the proteasome inhibitor MG132 (CHX + kaempferol + MG132). Under this condition, the half-life of BRG1 was extended to 73.47 hours (95% CI: 66.80–81.41 hours), which was even longer than that of the control group, indicating that MG132 effectively blocked the kaempferol-accelerated degradation. The goodness-of-fit (R^2^) values for the three curves were 0.9460, 0.9385, and 0.9778, respectively, indicating reliable fitting of the decay kinetics (Fig 5G). These results demonstrate that kaempferol promotes BRG1 degradation via the ubiquitin-proteasome pathway.

We next examined whether BRG1 degradation was associated with ubiquitination. Cells were pretreated with MG132 to block degradation of ubiquitinated proteins and then exposed to kaempferol. Immunoprecipitation of BRG1 followed by immunoblotting with an anti-ubiquitin antibody showed that kaempferol markedly increased BRG1 ubiquitination, and this effect was further enhanced in the presence of MG132, resulting in accumulation of ubiquitinated BRG1 species (Fig 5H). To explore the upstream mechanism, co-immunoprecipitation assays were performed using anti-BRG1 and anti-TRIM21 antibodies. Kaempferol treatment (50 μM, 24 hours) significantly enhanced the association between BRG1 and the E3 ubiquitin ligase TRIM21, with an enrichment increase of approximately 1.5-fold (*P* < 0.01; Fig 5I). Together, these findings support a model in which kaempferol promotes TRIM21-associated ubiquitination of BRG1, leading to proteasome-dependent degradation. Functionally, BRG1 knockdown markedly inhibited the proliferation and migration of colorectal cancer cells, as shown by EdU and migration assays (Fig 5J-L).

## Discussion

Kaempferol has recognized anticancer potential, but its clinical translation is limited by poor bioavailability and extensive metabolism [17,18]. In this study, we show that kaempferol exerts significant anti-colorectal cancer (CRC) activity in vivo and in vitro, and that its efficacy is determined not simply by total intracellular accumulation, but by the coordinated interplay among phase II metabolism, efflux transport, metabolite disposition, and target engagement. A central finding of this study is the distinct and seemingly paradoxical roles of UGT1A1 and UGT1A9 in regulating kaempferol activity. UGT1A1 knockdown increased intracellular, including nuclear, accumulation of kaempferol, yet unexpectedly reduced cytotoxicity, whereas UGT1A9 knockdown enhanced cellular sensitivity. These observations suggest that intracellular accumulation of the parent compound alone is insufficient to predict anti-CRC efficacy, and that the metabolic fate of kaempferol critically influences its pharmacological activity. Our data indicate that UGT1A1-mediated glucuronidation generates K-7-G, which retains substantial antiproliferative activity, whereas K-3-G, predominantly produced by UGT1A9, exhibits considerably weaker activity. Therefore, although UGT1A1 knockdown increases intracellular exposure to the parent compound, it simultaneously reduces the formation of the pharmacologically active metabolite K-7-G, thereby attenuating the overall antitumor effect. Notably, LC-MS/MS analysis demonstrated that the nuclear concentrations of both K-7-G and K-3-G were markedly lower than that of parent kaempferol, suggesting limited nuclear accessibility following glucuronidation. These findings imply that parent kaempferol likely remains the dominant regulator of nuclear targets such as BRG1, whereas K-7-G may contribute to anti-CRC activity through complementary or partially distinct mechanisms.

A similarly important divergence was observed for the two efflux transporters examined in this study. BCRP knockdown increased intracellular kaempferol accumulation and enhanced anti-CRC activity, consistent with the canonical role of BCRP in limiting intracellular drug exposure. In contrast, P-gP knockdown also increased intracellular accumulation, yet unexpectedly reduced the antitumor effect of kaempferol. Our data suggest that this apparent contradiction is driven by transporter-associated metabolic reprogramming. Specifically, P-gP knockdown increased UGT1A9 and modestly decreased UGT1A1, thereby shifting kaempferol metabolism away from K-7-G and toward K-3-G. Thus, greater total intracellular accumulation did not translate into improved efficacy because the composition of active versus weakly active species was altered. These findings further support that kaempferol efficacy depends on the coordinated balance between metabolism and transport rather than on accumulation alone.

The apparent discrepancy between the in vivo effective dose and the in vitro antiproliferative potency should also be interpreted cautiously. Plasma exposure to the parent compound alone does not fully reflect pharmacologically relevant exposure in colorectal tissues. Previous pharmacokinetic studies, together with our findings, suggest that kaempferol and its conjugates may achieve higher local exposure in the intestinal and colonic environment than would be predicted from plasma concentrations alone, partly because of tissue accumulation and enterohepatic recirculation [19]. Repeated daily administration is therefore likely to support sustained local exposure and persistent generation of active kaempferol-related species. In this context, the “bioavailability challenge” should not be viewed simply as an inability to achieve high plasma concentrations, but rather as a problem of optimizing tissue-relevant disposition, metabolic conversion, and intracellular retention of active species. From a translational perspective, modulation of phase II metabolism, inhibition of BCRP-mediated efflux, and formulation-based approaches may all represent potential strategies to improve kaempferol efficacy: (i) modulating UGT isoform balance to favor a metabolite profile enriched in K-7-G; (ii) selectively inhibiting BCRP-mediated efflux to improve intracellular retention of pharmacologically active kaempferol-related species; and (iii) formulation-based strategies, including nanoformulations or phospholipid-complex systems, to enhance solubility, local exposure, and intracellular delivery. At the same time, we explicitly acknowledge that these strategies should currently be regarded as mechanistically informed and hypothesis-generating future directions rather than immediate clinical recommendations. In particular, systemic co-administration of enzyme or transporter inhibitors may introduce challenges related to safety, selectivity, and drug–drug interactions, which will require careful pharmacokinetic and toxicological evaluation in future studies.

Another issue that should be noted is that the in vitro efficacy assays and mechanistic studies were conducted in different CRC cell lines. This design was intentional: HCT116 cells were used as a phenotypic model, whereas HT-29 and Caco-2 cells were selected for mechanistic analyses because of their relatively high endogenous expression of UGT1A1, UGT1A9, ABCG2, and Mdr1. Importantly, supplementary experiments confirmed that kaempferol also inhibited proliferation and migration in HT-29 and Caco-2 cells, supporting the cross-cell-line relevance of the major conclusions. Nevertheless, these conclusions should be interpreted as being supported across multiple CRC cell contexts rather than as evidence of complete uniformity among all cell lines.

To identify kaempferol-associated targets, we used SIP as an initial screening approach and then integrated proteomic, bioinformatic, and functional analyses. Among the candidate proteins identified, BRG1 emerged as the most compelling target after integrating subcellular localization, clinical relevance, SIP enrichment, network centrality, docking-based prioritization, and downstream functional validation (S3 A-H Fig). Kaempferol reduced BRG1 protein abundance, shortened its half-life, and this effect was almost completely reversed by MG132, indicating proteasome dependence. Ubiquitination assays further showed that kaempferol markedly increased BRG1 ubiquitination, and co-immunoprecipitation analyses identified enhanced association between BRG1 and the E3 ligase TRIM21 after kaempferol treatment. Together, these findings support a model in which kaempferol promotes TRIM21-associated ubiquitination and proteasomal degradation of BRG1, thereby contributing to suppression of CRC progression. At the same time, our data do not imply that nuclear targeting is a universal property of kaempferol; rather, within the present dataset, many candidate proteins were nuclear-localized, and BRG1 was validated as a functionally relevant nuclear target in CRC cells. This finding is particularly significant, given BRG1’s established role as an autophagy checkpoint regulator implicated in the pathophysiology of inflammatory conditions such as colitis [20].

In summary, this study shows that kaempferol exerts anti-CRC activity through a mechanism shaped by the coordinated interplay between phase II metabolism, efflux transport, metabolite disposition, and nuclear target engagement. UGT1A1, UGT1A9, BCRP, and P-gP differentially regulate the balance between active and weakly active kaempferol-related species, while BRG1 represents a functionally relevant nuclear target degraded through a ubiquitin-proteasome-dependent pathway. These findings provide a mechanistic framework for understanding kaempferol pharmacology in CRC and a basis for future metabolism-, transporter-, or formulation-guided optimization strategies.

## Conclusion

Our findings reveal that UGT1A1/1A9 and BCRP/P-gP critically regulate kaempferol’s intracellular disposition and anti-CRC activity. Kaempferol efficacy depends on the interplay of metabolism, efflux, metabolite composition, and target engagement, not simply total accumulation. BRG1 was identified as a nuclear target that undergoes ubiquitin-proteasome degradation upon kaempferol treatment (S4 Fig). These findings provide a mechanistic basis and rationale for improving kaempferol efficacy in CRC by modulating metabolism, retention, and target engagement.

## Supporting information

S1 Fig The pharmacodynamic assay of kaempferol. (A) Effect of kaempferol on organ weights in mice. (B) SRB assay for the effect of kaempferol on proliferation of HT29 and Caco2 cells. (C) Transwell assay for the effect of kaempferol on migration of HT29 cells. (D) Wound healing assay for the effect of kaempferol on migration of Caco2 cells.
(TIF)

S2 Fig The role of phase II metabolic enzymes in the inhibitory effect of kaempferol on CRC cell viability. (A) Western blot detection of UGT1A9 protein expression in HT-29 and Caco2 cells. (B) SRB assay showing the effect of UGT1A1 or UGT1A9 knockdown on the 24 h anti-proliferative activity of kaempferol in HT-29 cells. (C) SRB assay showing the effect of UGT1A1 or UGT1A9 knockdown on the 24 h anti-proliferative activity of kaempferol in Caco-2 cells. (D) SRB assay showing the effect of UGT1A1 or UGT1A9 knockdown on the 24 h anti-proliferative activity of kaempferol in HT-29 cells. (E) SRB assay showing the effect of UGT1A1 or UGT1A9 knockdown on the 24 h anti-proliferative activity of kaempferol in Caco-2 cells. (F) LC/MS detection of intracellular contents of kaempferol-7-O-glucuronide (K-7-G), kaempferol-3-O-glucuronide (K-3-G), and kaempferol in HT-29 and Caco2 cells after 24 h treatment with kaempferol.
(TIF)

S3 Fig Survival prognostic and expression profiling analysis of key genes in colorectal cancer. (A) Kaplan-Meier survival analysis of overall survival according to CBR1 expression in colorectal cancer patients. (B) Kaplan-Meier survival analysis of overall survival according to FOLR1 expression in colorectal cancer patients. (C) Kaplan-Meier survival analysis of overall survival according to AUH expression in colorectal cancer patients. (D) Kaplan-Meier survival analysis of overall survival according to AKR1C3 expression in colorectal cancer patients. (E) Protein expression of CBR1 in normal colon tissues versus primary colorectal cancer tissues. (F) Protein expression of AUH in normal colon tissues versus primary colorectal cancer tissues. (G) Protein expression of AKR1C3 in normal colon tissues versus primary colorectal cancer tissues. (H) mRNA expression of FOLR1 in normal colon tissues versus primary colorectal cancer tissues.
(TIF)

S4 Fig Graphical Abstract. (TIF)

S1 File Raw_images. (PDF)

S1 Table Q-PCR primer sequence. (DOCX)

S2 Table Up Differential proteins of SIP Samples Detected by mass spectrometry. (DOCX)

S3 Table Binding Energy of Kaempferol to Candidate Targets Predicted by Autodock. (DOCX)

S4 Table Binding Energy of Kaempferol to Candidate Targets Predicted by Ledock. (DOCX)

S5 Table Binding Energy of Kaempferol to Candidate Targets Predicted by Xdock. (DOCX)

## Abbreviations

CRC: colorectal cancer
LC-MS/MS: liquid chromatography-tandem mass spectrometry
LSCM: laser scanning confocal microscopy
SRB: sulforhodamine B
SIP: solvent-induced precipitation
KEGG: Kyoto Encyclopedia of Genes and Genomes
GO: Gene Ontology
QPCR: quantitative real-time polymerase chain reaction
BSA: bovine serum albumin
K-7-G: kaempferol-7-glucuronide
K-3-G: kaempferol-3-glucuronide
CETSA: cellular thermal shift assay
UGT1A1: UDP-glucuronosyltransferase family 1 member A1
UGT1A9: UDP-glucuronosyltransferase family 1 member A9
BCRP: breast cancer resistance protein
P-gP: P-glycoprotein
Mdr1: multidrug resistance protein 1
BRG1: Brahma-related gene 1
CHX: cycloheximide
PPI: protein-protein interaction
TCGA: The Cancer Genome Atlas
CPTAC: Clinical Proteomic Tumor Analysis Consortium
DMSO: dimethyl sulfoxide
PBS: phosphate-buffered saline
