# Supplementary material for: Orchestration of kaempferol nuclear translocation by phase ii metabolic enzymes and efflux transporters suppresses colorectal cancer
Source: PLoS One. 2026 Sep 15;21(9):e0355962. doi: 10.1371/journal.pone.0355962 (PMC13577484; doi:10.1371/journal.pone.0355962)

Fig 2, The Impact of Phase II Metabolic Enzymes on the Anti-Colorectal Cancer Efficacy of Kaempferol. C, Western Blot verification of UGT1A1 knockdown efficiency in transfected cells and statistical analysis.

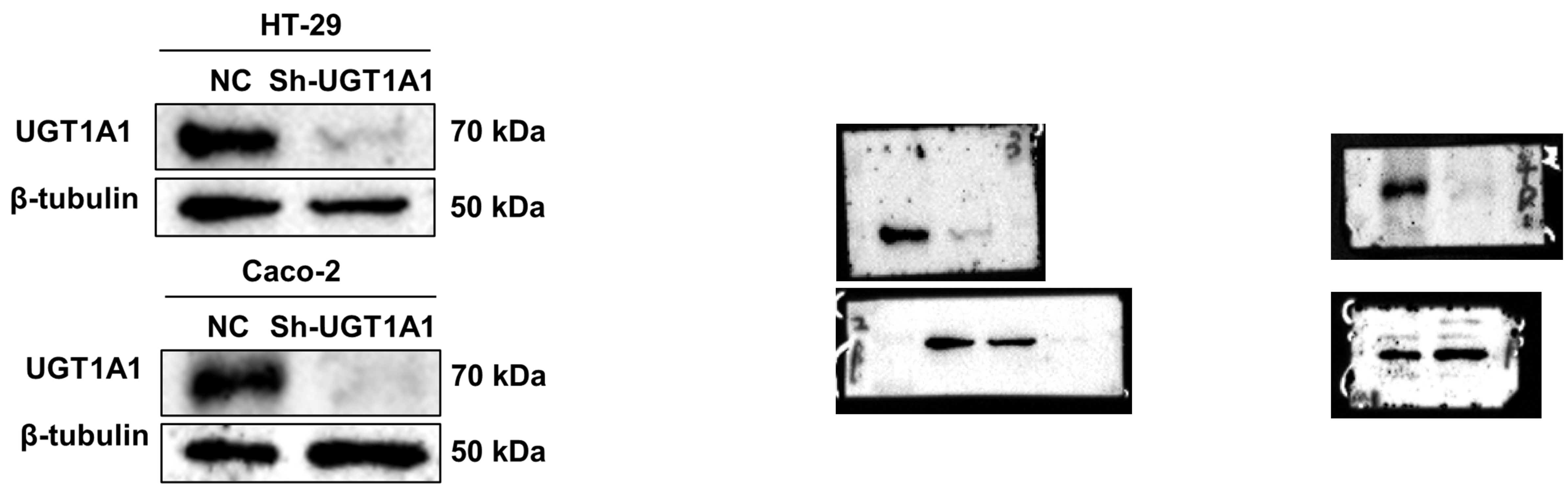

Fig 2, The Impact of Phase II Metabolic Enzymes on the Anti-Colorectal Cancer Efficacy of Kaempferol. D, Western Blot verification of UGT1A9 knockdown efficiency in transfected cells and statistical analysis.

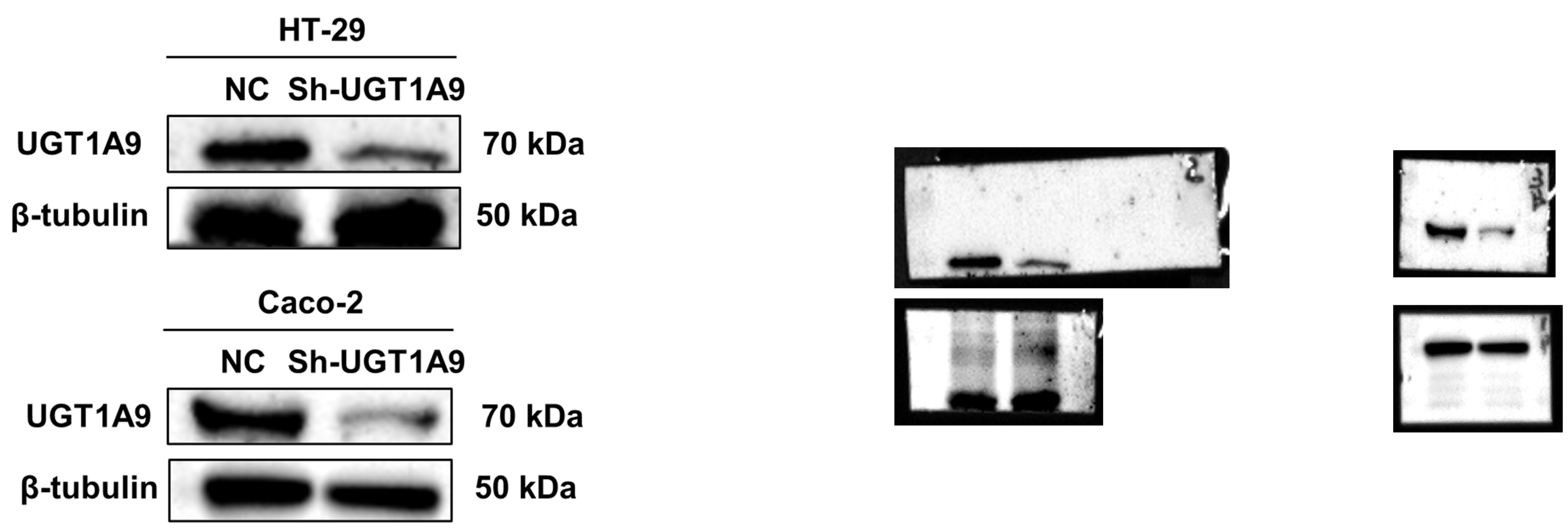

Fig 3, The Influence of Efflux Transporters on the Anti-Colorectal Cancer Efficacy of Kaempferol. C, Western Blot verification of ABCG2 knockdown efficiency in transfected cells and statistical analysis.

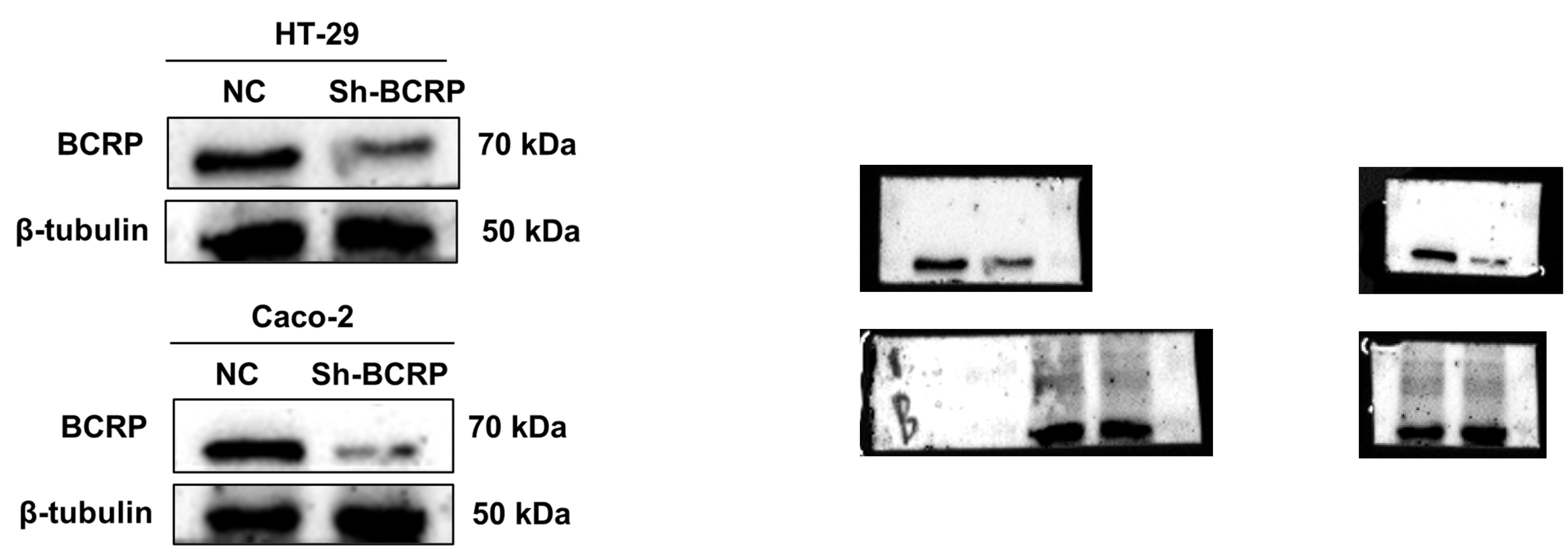

Fig 3, The Influence of Efflux Transporters on the Anti-Colorectal Cancer Efficacy of Kaempferol. D, Western Blot verification of Mdr1 knockdown efficiency in transfected cells and statistical analysis.

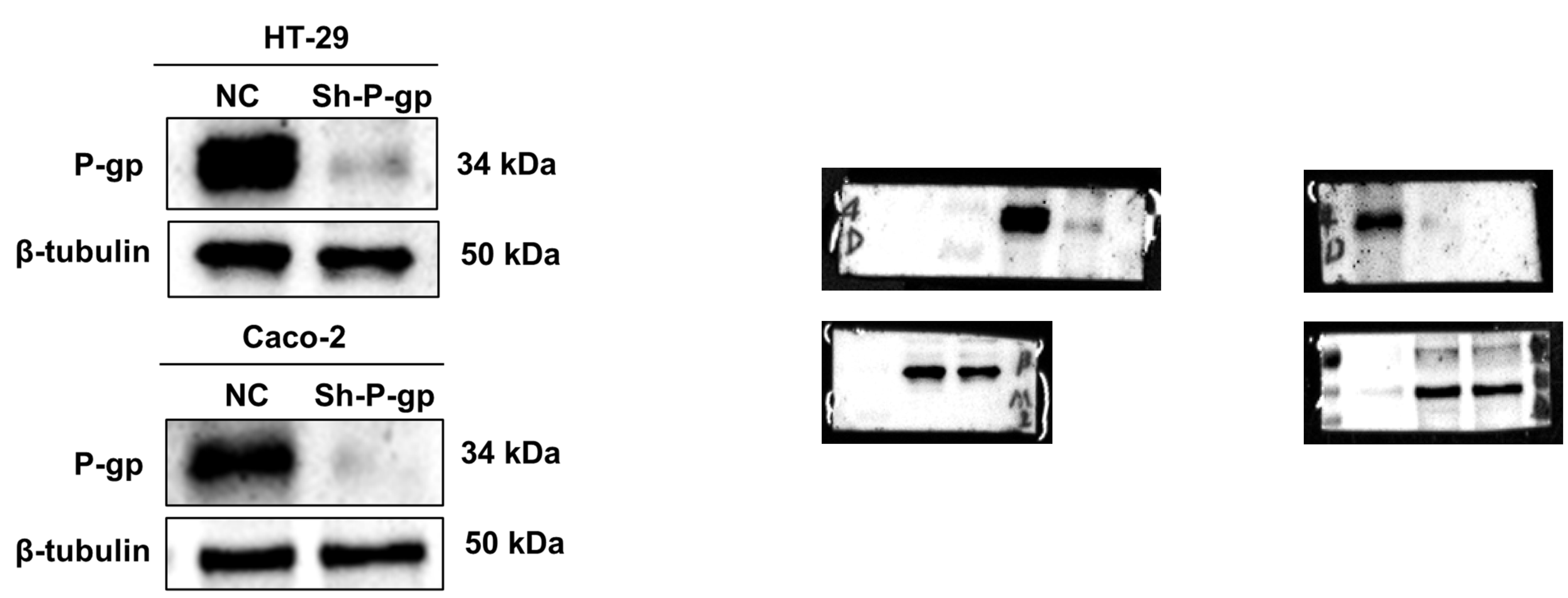

Fig 3, The Influence of Efflux Transporters on the Anti-Colorectal Cancer Efficacy of Kaempferol. K, P-gp knockdown upregulates UGT1A9 and downregulates UGT1A1 protein expression in Caco-2 and HT-29 cells.

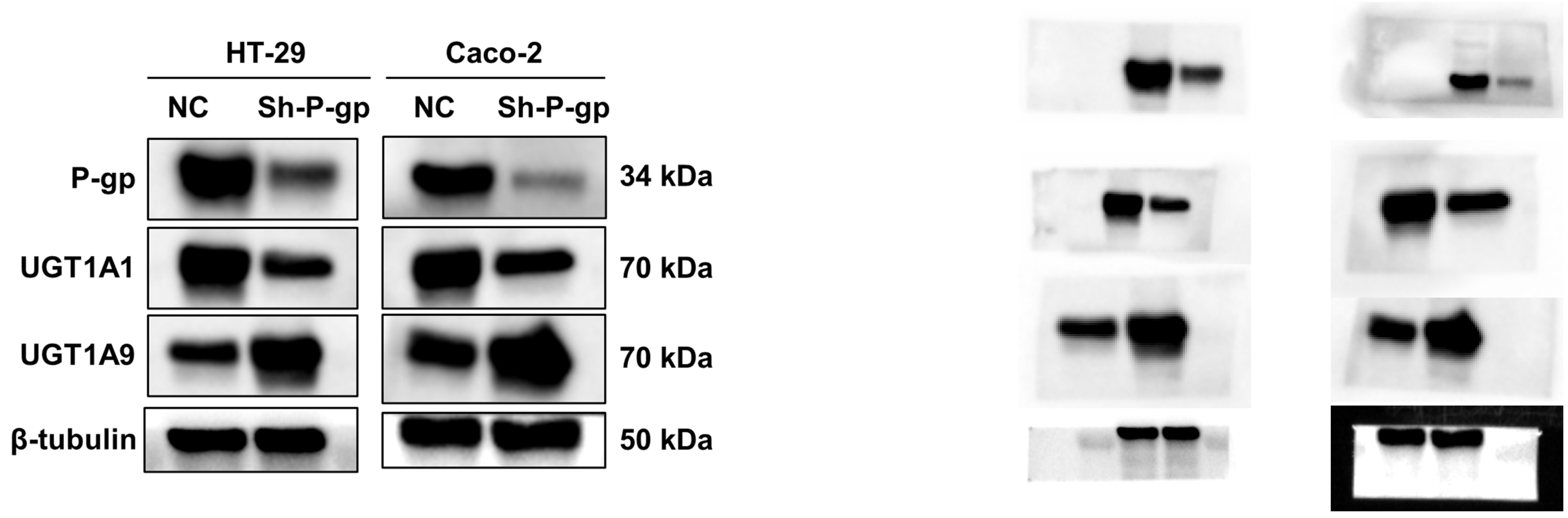

Fig 4, Identification of Kaempferol's Anti-Colorectal Cancer Targets. H, SIP Experiment Verification of the Binding Between Kaempferol and Potential Targets.

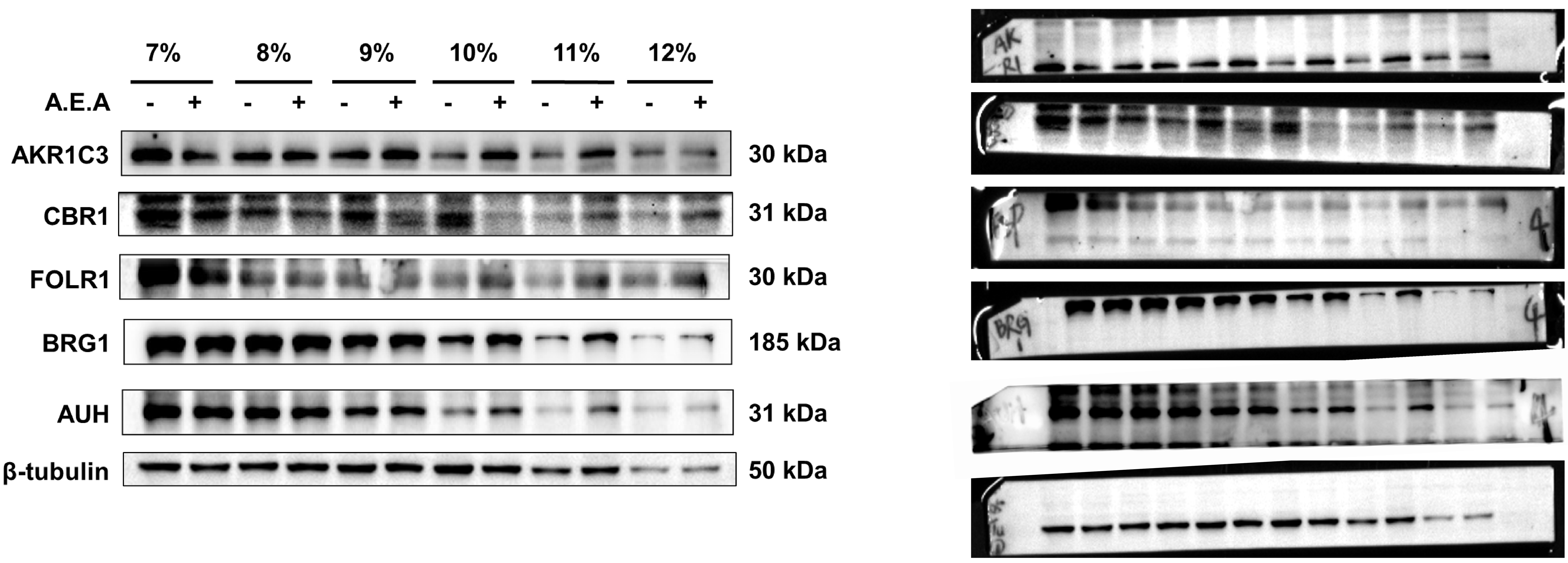

Fig 4, Identification of Kaempferol's Anti-Colorectal Cancer Targets. K, CETSA confirms the direct interaction between kaempferol and BRG1.

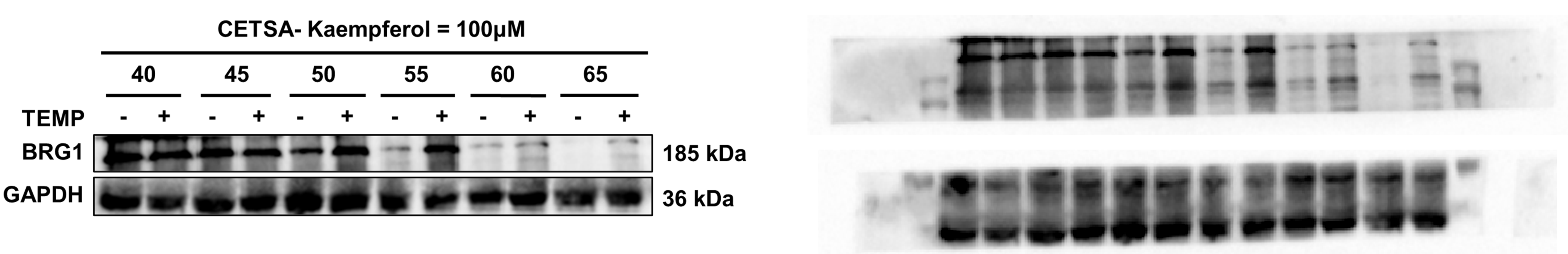

Fig 5, Kaempferol's effect on BRG1. F, Western Blot analysis of kaempferol's effect on BRG1 protein expression.

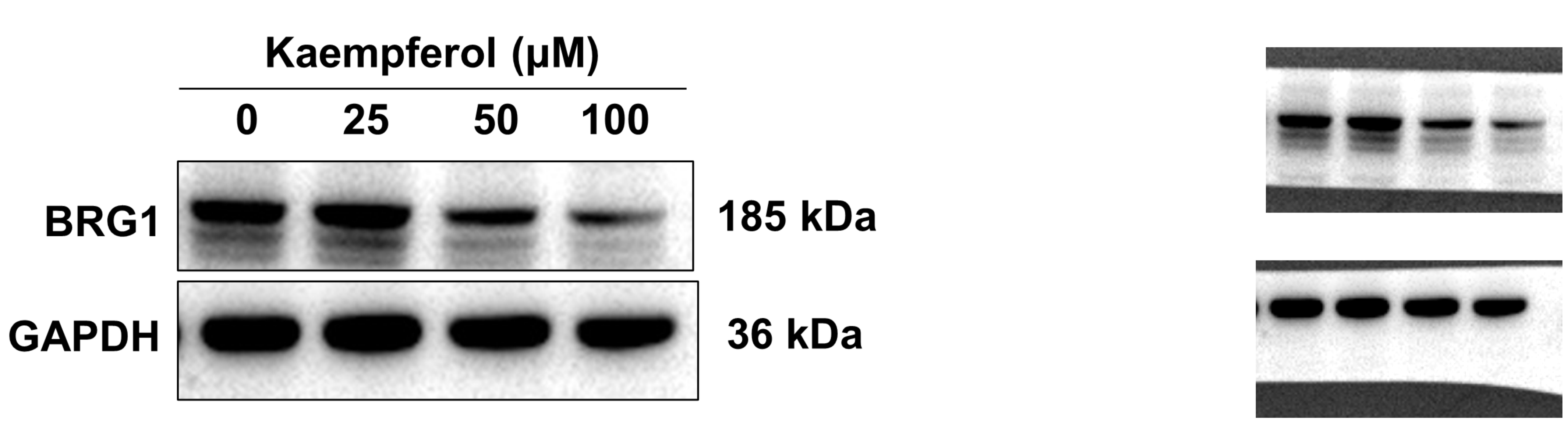

Fig 5, Clinical relevance of BRG1 and kaempferol-induced BRG1 degradation in colorectal cancer cells. G, Kaempferol reduces BRG1 protein stability via the proteasome pathway, as shown by CHX chase and MG132 rescue.

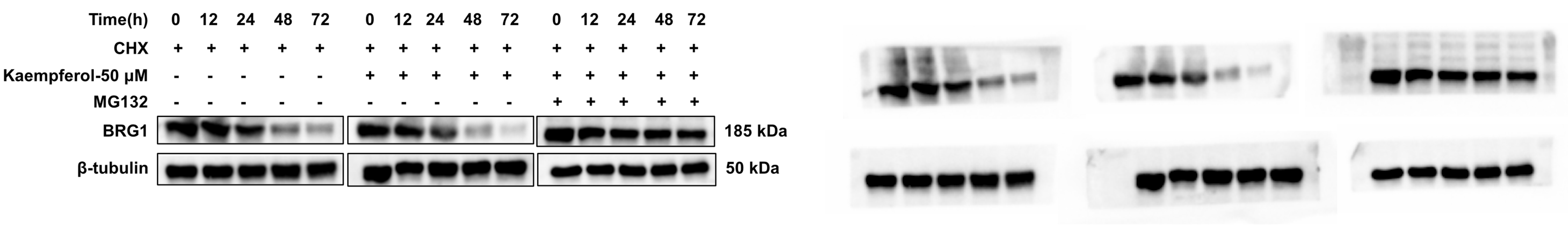

Fig 5, Clinical relevance of BRG1 and kaempferol-induced BRG1 degradation in colorectal cancer cells. H, Ubiquitination assay showing kaempferol-induced ubiquitination of BRG1 following immunoprecipitation.

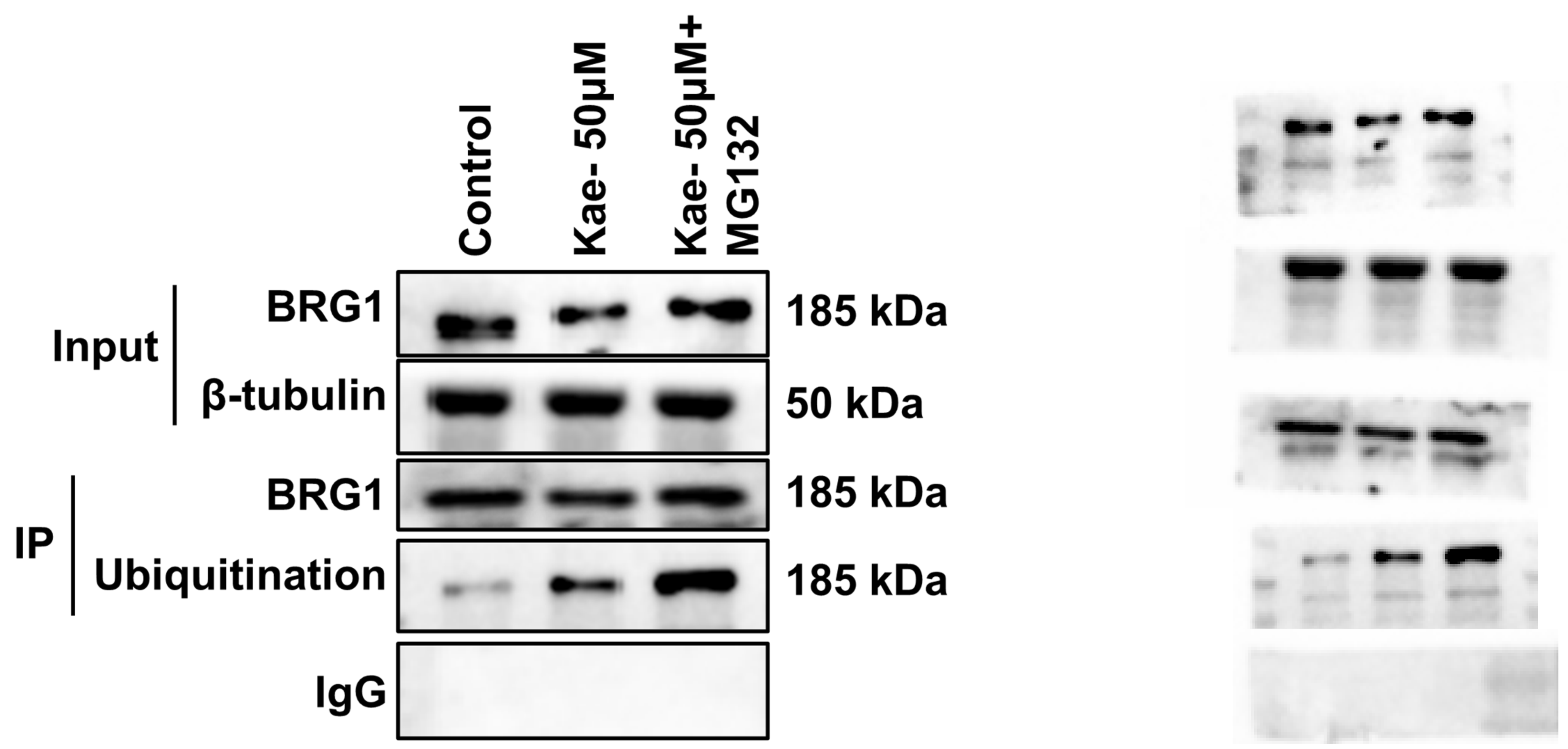

Fig 5, Clinical relevance of BRG1 and kaempferol-induced BRG1 degradation in colorectal cancer cells. I, Co-immunoprecipitation assay showing the association between BRG1 and TRIM21 after kaempferol treatment.

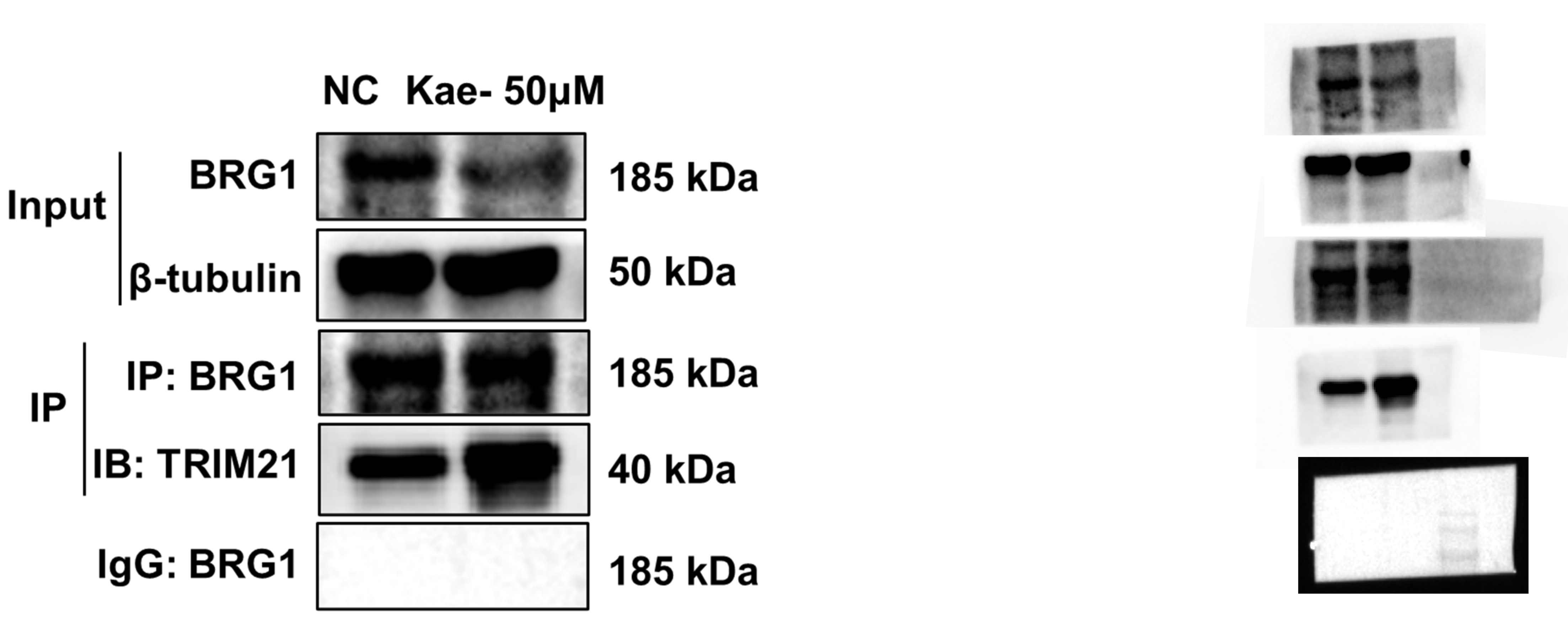

S2 Fig A, The protein expression of UGT1A9 in HT-29 and Caco2 cells was detected by Western blot.

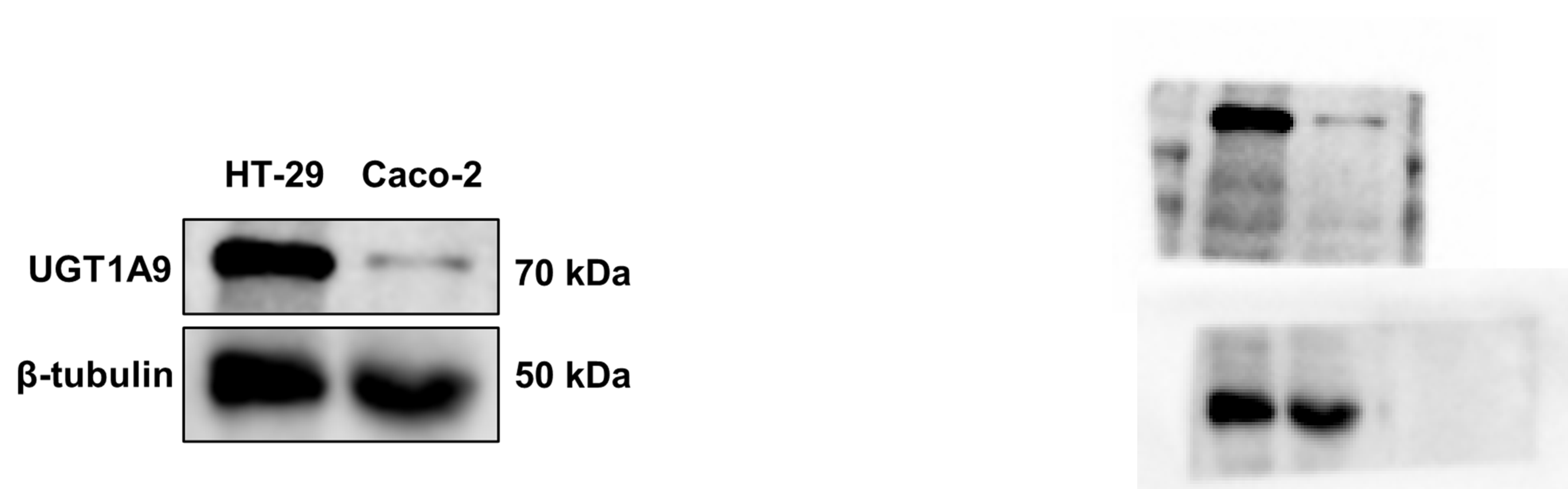

Supplement: Supplementary file 1 [file pone.0355962.s005.pdf]
