# Supplementary material for: Orchestration of kaempferol nuclear translocation by phase ii metabolic enzymes and efflux transporters suppresses colorectal cancer
Source: PLoS One. 2026 Sep 15;21(9):e0355962. doi: 10.1371/journal.pone.0355962 (PMC13577484; doi:10.1371/journal.pone.0355962)
Supplement: Supplementary file 2 [file pone.0355962.s006.docx]

S1 Table Q-PCR primer sequence

| **Primer Name** | **Primer Sequence (5’→3’)** |
| --- | --- |
| Human-UGT1A1-F | GTGTCCCATGCTGGGAAGAT |
| Human-UGT1A1-R | GAATGGCACAGGGTACGTCT |
| Human-UGT1A9-F | GACCGGGAGTTCAAGGCTTTT |
| Human-UGT1A9-R | CGTACTTGTGCTTTCCATTGAGC |
| Human-ABCG2-F | TTCTCCATTCATCAGCCTCG |
| Human- ABCG2-R | TGGTTGGTCGTCAGGAAGA |
| Human-Mdr1-F | GAGGGGATGGTCAGTGTTGATGG |
| Human-Mdr1-R | ATCGTGGTGGCAAACAATACAGGT |
| Human-GAPDH-F | CCATGGGGAAGGTGAAGGTC |
| Human-GAPDH-R | GCGCCCAATACGACCAAATC |
