# Supplementary material for: Orchestration of kaempferol nuclear translocation by phase ii metabolic enzymes and efflux transporters suppresses colorectal cancer
Source: PLoS One. 2026 Sep 15;21(9):e0355962. doi: 10.1371/journal.pone.0355962 (PMC13577484; doi:10.1371/journal.pone.0355962)
Supplement: Supplementary file 3 [file pone.0355962.s007.docx]

**S2 Table Up Differential proteins of SIP Samples Detected by mass spectrometry**

Protein Name

AXIN1

WIPF2

SMARCAD1

CBR1

COBLL1

SUN1

COPS8

SLC9A6

PPP1R13L

BIN1

RSF1

GPRIN1

KIAA1671

SNX18

IRAK1

MAP4K4

MYO9B

SCO1

PIR

C6orf132

MAGED1

AKR1C3

PRDX3

COPS3

CDC42EP4

CHCHD4

ESPN

C17orf80

SPTY2D1

LENG8

ZNF185

DDX51

FOLR1

ATAD2

HPCAL4

LCOR

LENG1

NFRKB

SPATS2L

NCAPD2

TNIP1

TNRC6A

MISP3

TCF7L2

PHF8

NES

CTDSPL2

MAP1S

POLE4

KHDC4

NFAT5

MRPL14

CAMSAP3

HS2ST1

USP36

GFER

AUH

NDUFA7

SMARCA4

BORCS5

ENPP5

H1-5

TCF20

EP400

DIAPH2

MAPT
