# Supplementary material for: Orchestration of kaempferol nuclear translocation by phase ii metabolic enzymes and efflux transporters suppresses colorectal cancer
Source: PLoS One. 2026 Sep 15;21(9):e0355962. doi: 10.1371/journal.pone.0355962 (PMC13577484; doi:10.1371/journal.pone.0355962)
Supplement: Supplementary file 4 [file pone.0355962.s008.docx]

**S3 Table Binding Energy of Kaempferol to Candidate Targets Predicted by Autodock**

| **Rank** | **Protein Name** | **Binding Energy（kcal/mol）** |
| --- | --- | --- |
| 1 | AKR1C3 | -9.7 |
| 2 | PIR | -8.2 |
| 3 | SLC9A6 | -8.1 |
| 4 | PHF8 | -8.1 |
| 5 | DDX51 | -7.7 |
| 6 | ATAD2 | -7.6 |
| 7 | GFER | -7.5 |
| 8 | HS2ST1 | -7.5 |
| 9 | USP36 | -7.5 |
| 10 | FOLR1 | -7.4 |
| 11 | SMARCAD1 | -7.4 |
| 12 | MAP4K4 | -7.2 |
| 13 | MAP1S | -7.2 |
| 14 | MYO9B | -7.0 |
| 15 | SMARCA4 | -7.0 |
| 16 | RSF1 | -7.0 |
| 17 | LENG8 | -6.9 |
| 18 | DIAPH2 | -6.8 |
| 19 | CAMSAP3 | -6.8 |
| 20 | CTDSPL2 | -6.8 |
| 21 | MAGED1 | -6.8 |
| 22 | PPP1R13L | -6.7 |
| 23 | CBR1 | -6.6 |
| 24 | COPS3 | -6.6 |
| 25 | SNX18 | -6.5 |
| 26 | IRAK1 | -6.5 |
| 27 | ESPN | -6.4 |
| 28 | COPS8 | -6.4 |
| 29 | COBLL1 | -6.4 |
| 30 | HPCAL4 | -6.3 |
| 31 | NFAT5 | -6.2 |
| 32 | AUH | -6.1 |
| 33 | H1-5 | -6.1 |
| 34 | AXIN1 | -6.0 |
| 35 | ENPP5 | -5.9 |
| 36 | SUN1 | -5.8 |
| 37 | SCO1 | -5.8 |
| 38 | KHDC4 | -5.6 |
| 39 | BIN1 | -5.5 |
| 40 | NFRKB | -5.5 |
| 41 | PRDX3 | -5.5 |
| 42 | MRPL14 | -5.2 |
| 43 | TCF20 | -5.2 |
| 44 | SPATS2L | -5.2 |
| 45 | ZNF185 | -4.9 |
