# Supplementary material for: Orchestration of kaempferol nuclear translocation by phase ii metabolic enzymes and efflux transporters suppresses colorectal cancer
Source: PLoS One. 2026 Sep 15;21(9):e0355962. doi: 10.1371/journal.pone.0355962 (PMC13577484; doi:10.1371/journal.pone.0355962)
Supplement: Supplementary file 5 [file pone.0355962.s009.docx]

**S4 Table Binding Energy of Kaempferol to Candidate Targets Predicted by Ledock**

| **Rank** | **Protein Name** | **Binding Energy（kcal/mol）** |
| --- | --- | --- |
| 1 | CBR1 | -6.62 |
| 2 | SLC9A6 | -6.41 |
| 3 | AKR1C3 | -6.36 |
| 4 | SNX18 | -6.05 |
| 5 | DDX51 | -5.94 |
| 6 | MYO9B | -5.71 |
| 7 | HS2ST1 | -5.71 |
| 8 | KHDC4 | -5.57 |
| 9 | AUH | -5.51 |
| 10 | ATAD2 | -5.29 |
| 11 | MAP1S | -5.17 |
| 12 | SMARCA4 | -5.14 |
| 13 | MAP4K4 | -5.12 |
| 14 | MRPL14 | -5.09 |
| 15 | ESPN | -5.04 |
| 16 | HPCAL4 | -4.97 |
| 17 | IRAK1 | -4.94 |
| 18 | NFAT5 | -4.93 |
| 19 | SMARCAD1 | -4.79 |
| 20 | ENPP5 | -4.73 |
| 21 | BIN1 | -4.72 |
| 22 | COPS8 | -4.58 |
| 23 | GFER | -4.47 |
| 24 | FOLR1 | -4.47 |
| 25 | DIAPH2 | -4.44 |
| 26 | CTDSPL2 | -4.44 |
| 27 | AXIN1 | -4.44 |
| 28 | NFRKB | -4.31 |
| 29 | LENG8 | -4.25 |
| 30 | USP36 | -4.24 |
| 31 | PHF8 | -4.19 |
| 32 | CAMSAP3 | -4.16 |
| 33 | H1-5 | -4.09 |
| 34 | COPS3 | -4.00 |
| 35 | MAGED1 | -3.97 |
| 36 | ZNF185 | -3.96 |
| 37 | PRDX3 | -3.9 |
| 38 | TCF20 | -3.79 |
| 39 | SPATS2L | -3.72 |
| 40 | PPP1R13L | -3.71 |
| 41 | COBLL1 | -3.51 |
| 42 | RSF1 | -3.48 |
| 43 | SCO1 | -3.44 |
| 44 | PIR | -3.41 |
| 45 | SUN1 | -3.20 |
