# Supplementary material for: Orchestration of kaempferol nuclear translocation by phase ii metabolic enzymes and efflux transporters suppresses colorectal cancer
Source: PLoS One. 2026 Sep 15;21(9):e0355962. doi: 10.1371/journal.pone.0355962 (PMC13577484; doi:10.1371/journal.pone.0355962)
Supplement: Supplementary file 6 [file pone.0355962.s010.docx]

**S5 Table Binding Energy of Kaempferol to Candidate Targets Predicted by Xdock**

| **Rank** | | **Protein Name** | **Binding Energy（kcal/mol）** |
| --- | --- | --- | --- |
| 1 | CBR1 | | -9.317 |
| 3 | AKR1C3 | | -8.201 |
| 4 | SNX18 | | -8.085 |
| 5 | DDX51 | | -8.081 |
| 6 | FOLR1 | | -7.789 |
| 7 | SMARCA4 | | -7.724 |
| 8 | SLC9A6 | | -7.633 |
| 9 | KHDC4 | | -7.537 |
| 10 | MAP4K4 | | -7.440 |
| 11 | RSF1 | | -7.372 |
| 2 | GFER | | -7.216 |
| 12 | MYO9B | | -7.190 |
| 13 | ENPP5 | | -7.151 |
| 14 | IRAK1 | | -6.974 |
| 15 | ATAD2 | | -6.925 |
| 16 | DIAPH2 | | -6.819 |
| 18 | MAP1S | | -6.789 |
| 19 | SUN1 | | -6.618 |
| 20 | MRPL14 | | -6.507 |
| 21 | AUH | | -6.375 |
| 22 | PIR | | -6.300 |
| 23 | HS2ST1 | | -6.280 |
| 24 | CAMSAP3 | | -6.250 |
| 25 | BIN1 | | -6.235 |
| 26 | TCF20 | | -6.233 |
| 17 | SMARCAD1 | | -6.809 |
| 27 | PPP1R13L | | -6.151 |
| 28 | USP36 | | -6.116 |
| 29 | HPCAL4 | | -6.096 |
| 30 | PHF8 | | -6.026 |
| 31 | NFAT5 | | -5.912 |
| 32 | COPS3 | | -5.816 |
| 33 | LENG8 | | -5.657 |
| 34 | SCO1 | | -5.652 |
| 35 | AXIN1 | | -5.616 |
| 36 | COPS8 | | -5.599 |
| 37 | PRDX3 | | -5.486 |
| 38 | ZNF185 | | -5.455 |
| 39 | NFRKB | | -5.367 |
| 40 | ESPN | | -5.364 |
| 41 | CTDSPL2 | | -5.818 |
| 42 | COBLL1 | | -5.295 |
| 43 | H1-5 | | -5.032 |
| 44 | MAGED1 | | -5.016 |
| 45 | SPATS2L | | -4.370 |
